# NAD Kinases: Metabolic Targets Controlling Redox Co-enzymes and Reducing Power Partitioning in Plant Stress and Development

**DOI:** 10.3389/fpls.2018.00379

**Published:** 2018-03-23

**Authors:** Bin-Bin Li, Xiang Wang, Li Tai, Tian-Tian Ma, Abdullah Shalmani, Wen-Ting Liu, Wen-Qiang Li, Kun-Ming Chen

**Affiliations:** State Key Laboratory of Stress Biology for Arid Areas, College of Life Sciences, Northwest A&F University, Yangling, China

**Keywords:** NAD kinases, calcium/calmodulin, enzymatic property, functional mechanism, pyridine nucleotides, reactive oxygen species, stress tolerance, plants

## Abstract

NAD(H) and NADP(H) are essential co-enzymes which dominantly control a number of fundamental biological processes by acting as reducing power and maintaining the intracellular redox balance of all life kingdoms. As the only enzymes that catalyze NAD(H) and ATP to synthesize NADP(H), NAD Kinases (NADKs) participate in many essential metabolic reactions, redox sensitive regulation, photosynthetic performance and also reactive oxygen species (ROS) homeostasis of cells and therefore, play crucial roles in both development and stress responses of plants. NADKs are highly conserved enzymes in amino acid sequences but have multiple subcellular localization and diverse functions. They may function as monomers, dimers or multimers in cells but the enzymatic properties in plants are not well elucidated yet. The activity of plant NADK is regulated by calcium/calmodulin and plays crucial roles in photosynthesis and redox co-enzyme control. NADK genes are expressed in almost all tissues and developmental stages of plants with specificity for different members. Their transcripts can be greatly stimulated by a number of environmental factors such as pathogenic attack, irritant applications and abiotic stress treatments. Using transgenic approaches, several studies have shown that NADKs are involved in chlorophyll synthesis, photosynthetic efficiency, oxidative stress protection, hormone metabolism and signaling regulation, and therefore contribute to the growth regulation and stress tolerance of plants. In this review, the enzymatic properties and functional mechanisms of plant NADKs are thoroughly investigated based on literature and databases. The results obtained here are greatly advantageous for further exploration of NADK function in plants.

## Introduction

NAD kinase (nicotinamide adenine dinucleotide kinase, NADK) is the only known enzyme that generates NADP(H) by phosphorylating NAD(H) in almost all living organisms (Gerdes et al., 2002; Mori et al., 2005a; Grose et al., 2006). As the major producer of NADP(H), NADK plays vital roles in maintaining the balance between NAD(H) and NADP(H) in NADP(H)-based cellular metabolic pathways (Kawai and Murata, 2008; Ying, 2008). In addition, NADK was found to be tightly related to protection against oxidative damage and plays crucial roles in cell survival and environmental stress tolerance both in animals and plants (Chai et al., 2005, 2006; Pollak et al., 2007b; Singh et al., 2007; Hashida et al., 2009; Shi et al., 2009; Gray et al., 2012; Petriacq et al., 2012, 2013, 2016). The number of NADK isoforms varies depending on the species. Archaea and Eubacteria usually have only one NADK, but most eukaryotes have multiple NADKs (Grose et al., 2006), and at least three NADK enzymes are found in plants (Li et al., 2014). These NADKs exist in different subcellular compartments including cytosol, chloroplasts, mitochondria and/or peroxisomes (Outten and Culotta, 2003; Shi et al., 2005; Pollak et al., 2007b; Kawai and Murata, 2008; Waller et al., 2010; Ohashi et al., 2012; Wang et al., 2016), showing their diverse functions. Recently, we found that the expression patterns of NADK family genes varied significantly with tissue and development specificity and environment differences (Li et al., 2014; Wang et al., 2016), further implying the functional diversity and divergence of NADKs in plants. However, the roles of NADKs and their functional mechanisms in plant growth and development are still under investigation.

## Discovery of plant NADKs

Research on NADK has been going on for more than half a century. In the early stage, NADK attracted attention due to its production of NADP playing a key role in photosynthesis and then its activity was examined in extracts of different higher plants. Yamamoto (1966) first detected the activity of NADK in the soluble portion of cell components of spinach leaves. Then, NADK was purified from pea (Muto and Miyachi, 1977) and wheat (Muto, 1982), respectively. Later, the activity of NADK was examined in corn (Dieter and Marme, 1984). During the researching on NADK, one of the significant findings was the discovery of a requirement for the calcium (Ca^2+^)-modulated protein, calmodulin (CaM). Muto and Miyachi (1977) found that purification of NADK from pea seedlings by DEAE-cellulose column chromatography resulted in loss of activity due to the dissociation of an activator. Then, Anderson and Cormier (1978) proved that CaM could act as the activator to activate the activity of Ca^2+^/CaM dependent NADK in pea. Later, Simon et al. (1982) found that both Ca^2+^/CaM-dependent and Ca^2+^/CaM-independent NADKs exist in pea seedlings. Since NADK was the first CaM-regulated enzyme discovered in plants, NADK was always used as a vehicle to examine the activity of CaM. At the beginning of this century, the first NADK gene (*ppnk*) was cloned in *Micrococcus flavus* (Kawai et al., 2000), thereafter, NADK genes were identified and cloned in various organisms (Table 1).

**Table 1:** The enzymatic properties of NADKs in different organisms.

**Species**	**Gene**	**Accession No**.	**Substrate**	**Phosphate donor**	**Multimerization**	**MW (subunits)**	***K_*m*_* (mM)**	***V_*max*_***	**Subcellular localization**	**References**
*Mycobacterium tuberculosis* H37Rv	*ppnk*	Z98268-16	NAD	ATP or poly(P)	Tetramer	35 kDa	ATP: 1.80 Poly(P)_4_: 1.60	ATP: 1.40 A Poly (P)_4_: 1.60 A	–	Kawai et al., 2000
*Micrococcus flavus*	*ppnk*	–	NAD	ATP or poly(P)	Dimer	34 kDa	ATP: 0.13 Poly(P)_4_: 1.04	ATP: 1.09 A Poly (P)_4_: 1.58 A	–	Kawai et al., 2000
*Escherichia coli* MG1655	*yfjB*	D90888-18	NAD	ATP or other nucleoside triphosphates	Hexamer	30 kDa	ATP:2.50 NAD: 2.00	–	–	Kawai et al., 2001a
*Archaeoglobus fulgidus*	*Afnk*	GI NO. 2650718	NAD	ATP	Tetramers or dimers	–	–	–	–	Liu et al., 2005
*Synechocystis* sp*. PCC 6803*	*SLr0400*	NP_441852	–	–	–	34 kDa	–	–	–	Gao and Xu, 2012
	*SLl1415*	NP_441342	–	–	–	34 kDa	–	–	–	Gao and Xu, 2012
*Fusarium oxysporum*	*FoNADHK*	FOXG_01647	NADH	ATP	Homodimer	72 kDa	ATP: 2.59 NADH: 0.13	–		Panagiotou et al., 2008
*Salmonella enterica*	*NadK*	16421206	NAD	ATP and other nucleoside triphosphates	Dimers and tetramers	36 kDa	ATP:2.7± 0.2 NAD: 2.1 ± 0.14	–	–	Grose et al., 2006
*Saccharomyces cerevisiae*	*UTR1*	NM_001181707	NAD	ATP, dATP, or CTP	Hexamer	60 kDa	ATP: 0.60 NAD: 0.50 NADH: 3.9	ATP: 1.2 B NAD: 1.2 B NADH: 3.5 B	Cytosol	Kawai et al., 2001b
	*POS5*	NM_001184002	NADH	ATP	–	46.3 kDa	–	–	Mitochondrial matrix	Outten and Culotta, 2003
	*YEF1*	NM_001178856	NADH	ATP	Homooctamer	60 kDa	ATP: 0.17 NAD: 1.9 NADH: 2.0	NADH: 1.9 B NAD: 1.7 B	Cytosol	Shi et al., 2005
*Strongylocentrotus purpuratus*	*SpNADK1*	ABY58956	NAD	ATP	–	–	NAD: 0.54 ATP: 0.397	2.27 D	–	Love et al., 2015
	*SpNADK2*	ABY58957	NAD	ATP	–	–	NAD:0.212 ATP: 0.263	1.38 D	–	Love et al., 2015
*Xenopus tropicalis*	*XtNADK*	IMAGE clone no. 7003620	NAD	ATP	–	–	NAD:0.756 ATP: 1.586	2.66 D	–	Love et al., 2015
*Pyrococcus horikoshii*	*PH1074* (*PhNADK*)	AB055976	NAD	GTP, CTP, UTP, ITP, and ATP/poly (P)	Tetramer	37 kDa	poly[27]: 0.30 ATP: 0.40	–	–	Sakuraba et al., 2005
*Corynebacterium glutamicum*	*CljPpnK*	1019388	NAD	ATP or poly(P)	Homotetramer	35.8 kDa	NAD: 4.02 ATP: 1.95	–	–	Shi et al., 2012
	*ClPpnK*	–	NAD	ATP or poly(P)	Homotetramer	35.8 kDa	NAD: 1.40 ATP: 2.12	–	–	Shi et al., 2012
*Aspergillus nidulans*	*AnNADHK*	AN8837	NADH	ATP	–	49 kDa	–	–	–	Panagiotou et al., 2009
*Sphingomonas* sp. A1	*SNADK*	AB127931	NAD	ATP or other nucleoside triphosphates	Homodimer	32 kDa	ATP: 1.92 NAD:0.43	–	–	Ochiai et al., 2004
*Thermococcus kodakarensis*	*ANKtk*	TK2124	NADH	–	–	40 kDa	–	–	–	Jia et al., 2010
*Bacillus subtilis*	*BsNADK*	C663_1189	NAD	poly(P), ATP, or other nucleoside triphosphates	Dimer	30 kDa	NAD: 1.0 ± 0.09	NAD: 2.6 ± 0.075 C ATP: 0.65 ± 0.08 C poly(P): 0.032 ± 0.09 C	–	Garavaglia et al., 2003
*Homo sapiens*	*C5orf33* (*MNADK*)	NM_001085411	NAD	ATP or poly(P)	Homodimer	45 kDa	ATP: 1.7 ± 0.3 NAD: 0.022 ± 0.001	0.091 ± 0.001 C	Mitochondria	Ohashi et al., 2012; Zhang, 2013
	*HuNADK*	NM_001198993	NAD	ATP	Homotetramer	49 kDa	ATP:3.3 NAD:0.54	6.7 D	Cytosol	Lerner et al., 2001; Pollak et al., 2007b
*Arabidopsis thaliana*	*AtNADK1*	AT3G21070	NAD	UTP or ATP	–	58.2 kDa	NAD: 0.52± 0.03 Mg^2+^·ATP: 0.73 ± 0.040	Mg^2+^·ATP: 11.1 ± 0.61 C	Cytosol	Turner et al., 2004; Waller et al., 2010
	*AtNADK2*	AT1G21640	NAD	UTP or ATP	–	109.2 kDa	NAD: 0.43 ± 0.017 Mg^2+^·ATP: 0.74 ± 0.012	Mg^2+^·ATP: 14.3 ± 0.30 C	Chloroplasts	Turner et al., 2004; Chai et al., 2005; Waller et al., 2010
	*AtNADK3*	AT1G78590	NADH	ATP,UTP, GTP and CTP	Dimer	35 kDa	ATP:0.062 Mg^2+^:1.16 NAD:2.39 NADH:0.042	ATP:39.5 ± 0.9 C Mg^2+^:40.3 ± 1.2 C NAD:23.2 ± 0.6 C NADH:41.2 ± 0.9 C	Peroxisomes	Turner et al., 2005; Waller et al., 2010
*Triticum aestivum*	*TaNADK1*	KP165048	–	–	–	–	–	–	Cytosol	Wang et al., 2016
	*TaNADK2*	KP165049	–	–	–	–	–	–	Cytosol	Wang et al., 2016
	*TaNADK3*	KP165050	–	–	–	–	–	–	Chloroplasts	Wang et al., 2016
	*TaNADK4*	KP165051	–	–	–	–	–	–	Peroxisomes	Wang et al., 2016

*NADKs were identified and cloned from different organisms. The enzymatic properties of these NADKs were summarized here, including the preferable substrates, phosphate donors, multimerization, subunit molecular weight, K_m_ and V_max_. To keep consistent with the original data, the units of V_max_ were presented as follows: A, μmol/unit/h; B, mM min^−1^ U^−1^; C, U/mg; D, μm∙(min∙mg)^−1^*.

In most prokaryotic organisms like *Micrococcus flavus* and *Escherichia coli* (*E. coli*), there is only one NADK responsible for the synthesis of NADP (Kawai et al., 2000, 2001a). However, multiple NADKs were identified in yeast and higher plant cells. *Saccharomyces cerevisiae* (*S. cerevisiae*) has three NADKs (POS5, UTR1 and YEF1) (Kawai et al., 2001b; Outten and Culotta, 2003; Shi et al., 2005), whereas higher plants usually have more than three. In *Arabidopsis thaliana*, three NADK genes, namely *AtNADK1, AtNADK2*, and *AtNADK3*, were cloned and examined (Turner et al., 2004, 2005). Recently, we identified eleven NADK genes in *Triticum aestivum*, which encode four NADK isoforms, named TaNADK1, TaNADK2, TaNADK3, and TaNADK4, respectively (Wang et al., 2016). We also performed a comparative genomic analysis of NADK family genes within eight supergroup plantae and classified the gene family into four subfamilies according to their phylogenetic relationship and structure, but the NADKs from land plants are mainly fallen into subfamily I, II, and III (Li et al., 2014). Each subfamily has its own conserved regions in the NAD kinase domain. According to the domain composition, AtNADK1, TaNADK1 and TaNADK2 belong to subfamily I, AtNADK2 and TaNADK3 belong to subfamily II, whereas AtNADK3 and TaNADK4 were grouped to subfamily III. All identified NADKs retain specific motifs including the GGDG motif, NE/D motif and Gly-rich motif, which are considered as conserved motifs in NADKs (Labesse et al., 2002; Garavaglia et al., 2004; Raffaelli et al., 2004; Liu et al., 2005; Mori et al., 2005b). In addition, according to the domain composition, a model for the evolution of plant NADKs was proposed (Li et al., 2014). By this model, it was concluded that all NADKs originated from a common ancestor, which contained only the typical NAD kinase domain and existed in all living organisms from prokaryotic bacteria to eukaryotic angiosperms, and then gene fusion and exon shuffling after gene duplication contributed to the expansion and evolution of the NADK family in plants (Li et al., 2014).

## Structure and enzymatic properties of plant NADKs

NADK is considered as the sole enzyme that phosphorylates NAD to NADP. As shown in Table 1, different NADKs have their own preferable substrates between NAD and NADH with inorganic polyphosphate [poly(p)], ATP or other nucleoside triphosphates as their primary phosphate donors. Poly(P)/ATP-NADKs were found to be distributed throughout Gram-positive bacteria and Archaea, whereas ATP-specific NADKs were found to exist mainly in Gram-negative α- and γ-proteobacteria and eukaryotes (Nakamichi et al., 2013).

In *Arabidopsis thaliana*, as mentioned above, there are three identified NADKs, namely AtNADK1, AtNADK2 and AtNADK3 (Turner et al., 2004, 2005). All three NADKs can utilize ATP or UTP but not poly(p) as the phosphate donor for the phosphorylation of NAD/NADH. In addition, AtNADK3 also can utilize GTP and CTP as a phosphate donor (Turner et al., 2005). AtNADK1 and AtNADK2 can catalyze the phosphorylation of NAD, and AtNADK3 can catalyze the phosphorylation of NADH (Turner et al., 2004, 2005). Although the functions of these NADKs are well known, the structure of plant NADKs and their precise reaction mechanism remained speculative.

Several researches on bacterial NADKs may give the clue for further exploration of plant NADKs. Garavaglia et al. (2004) obtained two crystal structures of NADK in *Mycobacterium tuberculosis* by multiwavelength anomalous dispersion, which revealing that NADK exist as dimers or tetramers. Each subunit (Ppnk) of the crystal structures consists of an α/β N-terminal domain and a C-terminal 12-stranded β sandwich domain, connected by swapped β strands. The catalytic site is located in the long crevice that defines the interface between the domains. Given that the conserved GDDG motif of catalytic site, NADK was proposed to belong to a superfamily of kinases, which includes 6-phosphofructokinases, diacylglyceride kinases, and sphingosine kinases (Labesse et al., 2002). The strictly conserved motif GGDG has been demonstrated playing a key role in ATP binding.

**Figure 1:**
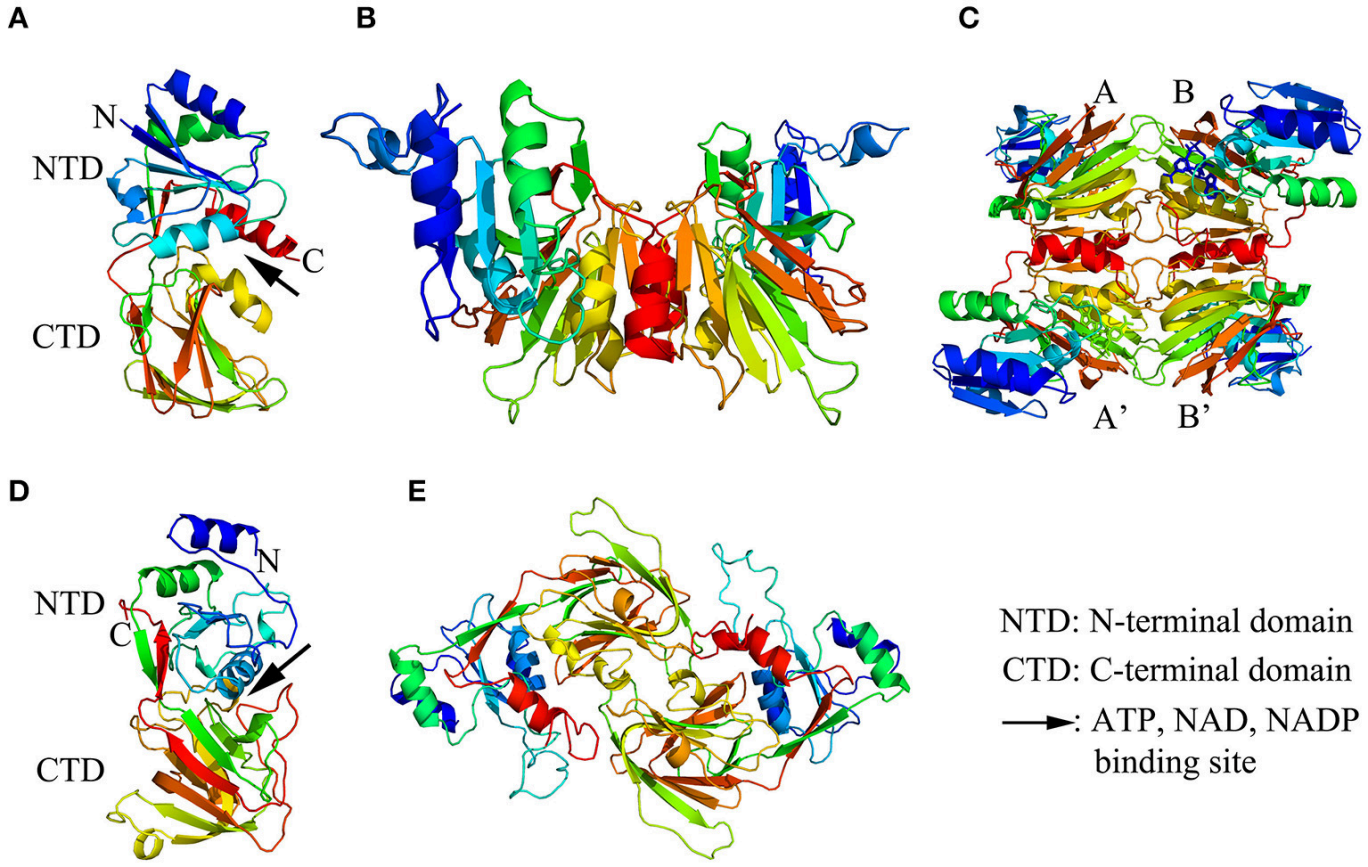
The structure models of NAD kinase. The structure models were constructed using a homology modeling method with the full-length amino acid sequences of a NAD kinase from *Archaeoglobus fulgidus* (Afnk) and a NADH kinase from *Arabidopsis thaliana* (AtNADK3). **(A–C)** showed the ribbon diagram of Afnk monomer, dimer, and tetramer, respectively. The four monomers in the Afnk tetramer were labeled with A, A', B, and B' as previously reported (Liu et al., 2005). **(D,E)** Illustrated the ribbon diagram of AtNADK3 monomer and dimer, respectively. Models were generated in Pymol and colored by rainbow from N terminus to C terminus.

The crystal structure of NADK in complex with ATP, NAD, or NADP was clarified by Liu et al. (2005) and Poncet-Montange et al. (2007) in *Archaeoglobus fulgidus* and *Listeria monocytogenes*, respectively, and a possible phosphate transfer mechanism for NADKs was proposed. Liu et al. (2005) found that the monomer structure of Afnk, a NADK from *Archaeoglobus fulgidus*, is composed of 6 α-helices and 16 β-strands that organized into two domains: an N-terminal domain (NTD) and a C-terminal domain (CTD) (as shown in Figure 1A). The overall fold of Afnk is similar to the Ppnk (a NADK from *Mycobacterium tuberculosis*) structure and NAD, NADP and ATP are all bound in the deep cleft (pointed out with a black arrow in Figure 1A) between NTD and CTD. The conversed GGDG loop forms hydrogen bounds with the phosphate group in the ATP-bound structure and the 2′ phosphate group in the NADP-bound structure. In addition, NAD and NADP shared an almost identical chemical environment, indicating that substrate NAD and product NADP shared the same binding site. This finding implied that the GGDG motif plays an important role in the phosphate transfer mechanism. Based on these observations, a model for NAD phosphorylation was proposed: NADK contains a dinucleotide-binding site composed of two subsites: the AMP-binding site (subsite A) and the nicotinamide binding site (subsite N). Before the phosphorylation, NAD binds to subsite A with its AMP portion and binds to subsite N with its nicotinamide ribose portion. During phosphorylation, the AMP portion of NAD is replaced by the AMP portion of ATP. After phosphorylation, the AMP portion of NADP binds back to subsite A and ADP is released. Poncet-Montange et al. (2007) proposed a similar model but with two differences. Firstly, both the phosphate group of the donor and the diphosphate group of the substrate are needed to chelate the catalytic di-cation magnesium (Mg^2+^). Secondly, the phosphate donor does not have to bind to subsite A during phosphorylation. Both models disclosed the probable mechanism of NAD phosphorylation.

According to the preference of NAD or NADH, NADKs can be divided into two types as NAD kinase and NADH kinase (NADHK). NAD kinase always chooses NAD as its primary substrate. A NADK, namely Ppnk, which was identified from *M. flavus* and *M. tuberculosis H37Rv*, can specifically and completely phosphorylate NAD to NADP, but could not phosphorylate NADH even in the presence of poly(P) or ATP (Kawai et al., 2000). POS5 is the first member of NADH kinase family identified from yeast, its NADH kinase activity increased about 50 fold compared to its NAD kinase activity (Outten and Culotta, 2003). Although NAD kinase and NADH kinase have different abilities to phosphorylate NAD or NADH, in some circumstances, they can transfer their ability from each other. Mori et al. (2005a) found that a single amino acid substitution leads to the molecular conversion of NAD kinase and NADH kinase. It was reported that conversion of Arg-293 of yeast POS5 into a His residue reduced the ratio of NADH kinase activity to NAD kinase activity, whereas simultaneous changing the Ala-330 and His-351 of human NAD kinase into Ser and Arg residues, respectively, significantly increased the ratio of the two enzymatic activities (Ando et al., 2011). Recently, Nakamichi et al. (2013) showed that ATP-specific NADKs can be conferred the ability to utilize poly(P) through a single amino acid substitution in *E. coli*. They successfully conferred γ-proteobacterial ATP-specific NADKs the ability to utilize poly(P) for the poly(p)-dependent mass production of NADP.

Moreover, numerous studies indicated that NADK functions as multimers. The multimerization of NADKs in different organisms were summarized in Table 1. In *Archaeoglobus fulgidus*, Afnk crystal structures exist as tetramer in the refolded Afnk protein (Afnk-NAD and Afnk-ATP structure) and as dimers in its NADP complex (Afnk-NADP structure) (Figures 1B,C; Liu et al., 2005). The tetramers included two types of dimers: the AA′ dimer and the AB dimer. The AA′ dimer involves extensive interactions between C-terminal domains, which is also found in the Ppnk crystal structure. Overall, the AA′ dimer is probably the most basic oligomeric state for NAD kinase. The AB dimer is involved in substrate binding, phosphate donor binding and ATP binding (Liu et al., 2005). Grose et al. (2006) reported that NADK in *Salmonella enterica* exists as an equilibrium mixture of dimers and tetramers, but it would entirely transform into tetramers when it was inhibited by NADH or NADPH.

In addition, NADK in different organisms usually functions as different multimer types. In *E. coli*, yjfB (NADK) functions as a hexamer with six 30 kDa subunits (Kawai et al., 2001a). In *S. cerevisiae*, the multimerization of two NADKs, namely UTR1 and YEF1, were examined. UTR1 exists as hexamer with six 60 kDa subunits while YEF1 is composed of ten similar 60 kDa subunits (Kawai et al., 2001b; Shi et al., 2005). In higher plants, Turner et al. (2005) found that an *A. thaliana* NADK, AtNADK3, exists as dimers by gel filtration study. The crystal structures of AtNADK3 monomers and dimers have been presented in Figures 1D,E, which were predicted with SWISS-MODEL (https://swissmodel.expasy.org/interactive). However, the enzymatic properties of NADKs in higher plants have not been well elucidated yet. Further studies are needed to clarify the enzymatic mechanisms of different NADKs in plants that consider both the paralogous members from the same species and the orthologous ones from different species.

## Biochemical regulation of the activity of NADKs in plants

### Ca^2+^/CaM involved regulation of NADK activity

Lots of regulators have been found playing important roles in regulating NADK activity. According to whether Ca^2+^/CaM is needed for NADK activation, NADKs can be divided into two types in plants, Ca^2+^/CaM dependent NADKs and Ca^2+^/CaM independent NADKs. Simon et al. (1984) found that in chloroplasts of pea seedlings, a Ca^2+^/CaM dependent NADK exists in the envelope of chloroplasts whereas a Ca^2+^/CaM independent NADK is found in the stroma of the organelle. Ca^2+^ and CaM are necessary for the Ca^2+^/CaM dependent NADKs. Roberts et al. (1986) reported that lycine-115 methylated CaMs activated an NAD kinase preparation to a maximal level that was at least 3–4-fold lower than the level of activation obtained with the corresponding unmethylated CaMs. Two tobacco transgenic plants, VU-1, which expressed a CaM that can be methylated at the lycine-115 residue, and VU-3, which expressed a CaM where the lycine-115 was substituted with arginine and was incapable of methylation, exhibited different growth behaviors (Roberts et al., 1992). VU-1 showed no difference compared to wild type, but VU-3 lines were characterized by decreased stem internode growth, reduced seed production, and reduced seed and pollen viability. A possible reason is that overexpressing VU-3 in plants led to an alteration in cellular metabolism due to the disruption of nicotinamide co-enzyme homeostasis (Roberts et al., 1992). Harding et al. (1997) found that VU-3 lines have increased NADPH levels and coincided both in timing and magnitude with development of the reactive oxygen species (ROS) burst. This result indicated that plant NADK may be a downstream target which potentiates ROS production by altering NAD(H)/NADP(H) homeostasis. Further study indicated that NADK is involved in the CaM signaling pathway meditated plant oxidative burst and the hypersensitive response (Harding and Roberts, 1998).

Multiple CaM isoforms with minor amino acid sequence differences have been identified in plants. Lee et al. (1995) found two kinds of CaMs (SCaM-1 and SCaM-4) from soybean which had different effects on the Ca^2+^/CaM dependent NADK purified from pea seedlings, one is highly conserved soybean SCaM-1 which could activate the NADK, but its divergent isoform SCaM-4 could not. By generating a series of chimeric SCaMs with exchanging functional domains between SCaM-1 and SCaM-4, they found that domain I of CaM plays a key role in differential activation of NADK and the residues Lys-30 and Glu-40 of SCaM-1 are critical for this function (Lee et al., 1997). Then, Cobb et al. (1999) found that the QNP sequence in a loop between EF hands I and II in CaM is necessary for NADK activation. In tobacco, it was found that three types of CaM isoforms differentially activate NADK activity, the activity was activated most effectively by type II, moderately by type I, but weakly by type III (Karita et al., 2004). More recently, it was proved that SCaM-1 and SCaM-4 could differentially regulate NADK activity, activated by SCaM-1 but inhibited by SCaM-4 because of their different responses to the Ca^2+^ signal (Walton et al., 2017).

The activity of NADK can be regulated by altering the activity of activator CaM, but how CaM regulates NADK remains unclear. In *Arabidopsis thaliana*, Turner et al. (2004) found that AtNADK2 can interact with CaM, and a 45-residue region within the N-terminal domain was identified as the Ca^2+^-dependent CaM binding domain, but the recombinant AtNADK2 was not responsive to CaM *in vitro*. Love et al. (2015) proposed a Ca^2+^/CaM involved animal NADP synthesis model. In this model, SpNADK2 could be directly regulated by Ca^2+^/CaM binding, and SpNADK1 could be phosphorylated by CaM-dependent protein kinase II. However, further studies are needed to clarify whether a similar mechanism is functioning in plants.

Since CaMs are effective activators for the Ca^2+^/CaM-dependent NADKs, inhibition of CaMs activity is also a strategy for the inhibition of NADK activity. A series of phytotoxins from the fungus *Guanomyces polytrix* have been shown to inhibit NADK activity by reducing the activity of CaM (Mata et al., 2003).

### The allosteric regulation mode of NADK

NADK can catalyze NAD or NADH and ATP to synthesize NADP or NADPH, but its activity can be affected by competitive substrates which contribute to the NADP(H)/NAD(H) homeostasis in cells. In spinach, NADH was found to be a potent competitive inhibitor of NADK and the *K*_*i*_ (competitive inhibition constants) was 1 × 10^−4^ M (Yamamoto, 1966). In addition, the activity of recombinant NADHK (AtNADK3) was inhibited by poly-P_i_. Mixed inhibition was observed regarding either ATP or NADH, indicating that poly-P_i_ may function at an allosteric site. The *K*_*i*_ and *K*_*i*_′ (uncompetitive inhibition constants) of the NADHK for ATP and NADH are 0.23 and 1.21 mM, and 0.51 and 2.86 mM, respectively (Turner et al., 2005).

NADKs from human or microbes were also regulated by effectors like NAD(H) and NADP(H), even though the specific regulator for each NADK is different. Purified human NADK, which was expressed in *E. coli*, exhibited the inhibitory effects of NADPH and NADH but not of NADP (Ohashi et al., 2011). In *E. coli* and *S. enterica*, NADH and NADPH were also found to be effective inhibitors to NADK (Zerez et al., 1987; Grose et al., 2006). It was reported that the activity of NADK can be inhibited by both NADP and NADPH in *Sphingomonas* sp. A1 (Ochiai et al., 2004) but only strongly inhibited by NADP in *Bacillus licheniformis, Bacillus subtilis* and *M. tuberculosis* (Zerez et al., 1986; Garavaglia et al., 2003; Raffaelli et al., 2004). In *M. tuberculosis* and *M. flavus*, however, the activity can be inhibited by a low concentration of NADPH (Kawai et al., 2000). NADK activity can also be inhibited by NAD analogs in eukaryotic organisms. In *S. cerevisiae*, it was found that the NADK activity of Utr1p was strongly inhibited by NADP, NADH and NADPH (Kawai et al., 2001b), whereas the activity of Yef1p was only slightly inhibited by NADH and NADPH (Shi et al., 2005). Recently, Paoletti et al. (2016) found that a series of NAD mimics such as 8-thioalkyladenosine derivatives, can act as competitive substrates to inhibit *Listeria monocytogenes* NADK1 and *Staphylococcus aureus* NADK as well as their human counterpart, implying a similar allosteric regulation mode of these NADKs. In some specific situations, the activity of NADK can also be inhibited through the interaction with other enzymes. For example, ANKtk, an ATP/NAD kinase from *Thermococcus kodakarensis* KOD1 can bind to oxidized NOXtk hexamers which can inhibit NADK catalytic activity (Jia et al., 2010). NOXtk is a NADH oxidase from *T. kodakarensis* KOD1, it can be inhibited by oxidation and both unoxidized and oxidized NOXtk can form dimers and hexamers (Jia et al., 2010). However, no more evidence was provided to further clarify the allosteric regulation of NADKs, particularly in plants.

## Multiple subcellular localizations of NADKs in plants

There is more than one NADK existing in eukaryotic cells, which can make them synthesize NADP in different subcellular compartments. In *S. cerevisiae*, both UTR1 and YEF1 are located in the cytosol, but POS5 is located in mitochondrial matrix (Kawai et al., 2001b; Outten and Culotta, 2003; Shi et al., 2005). In human cells, first NADK was discovered in 2001 and found to be located in cytosol (Lerner et al., 2001; Pollak et al., 2007b). More than 10 years later, another human NADK, namely MNADK, was identified in mitochondria and found to be responsible for *de novo* synthesis of NADP in this subcellular compartment (Ohashi et al., 2012; Zhang, 2013). In higher plants, NADK proteins also have multiple subcellular localizations. In *Arabidopsis thaliana*, it was found that AtNADK1 is located in the cytosol; AtNADK2 is in chloroplasts, but AtNADK3 is in peroxisomes (Turner et al., 2004, 2005; Chai et al., 2005; Waller et al., 2010). In wheat, we found that TaNADK1 and TaNADK2 are located in the cytosol, TaNADK3 is in chloroplasts but TaNADK4 in peroxisomes (Wang et al., 2016).

Mitochondria are one of the major producers for ROS as by-products from the reduction of O_2_ to H_2_O during oxidative phosphorylation of cells (Boveris, 1984) and NADPH is an important source of reduction power in the ascorbate-glutathione cycle (Halliwell-Asada cycle), which plays a key role in the ROS scavenging system (Mittler, 2002)—accordingly, the cells need a large amount of NADPH to deal with excess ROS production. In mammalian cells, except MNADK, mitochondrial NADP-dependent isocitrate dehydrogenase has been reported to be an important source of mitochondrial NADPH (Jo et al., 2001). In plant cells, mitochondrial NADP-specific isocitrate dehydrogenase was also identified to be responsible for mitochondrial NADPH production (McIntosh and Oliver, 1992), but no mitochondrial NADK has been identified in plant cells so far, even though an earlier report indicated that NADK activity was found in the outer mitochondrial membrane of corn cells (Dieter and Marme, 1984). Since specific NADKs exist in mitochondria of yeast and human cells for NADPH production (Outten and Culotta, 2003; Ohashi et al., 2012), it is interesting to know whether or not a certain NADK exists in mitochondria of plant cells.

**Figure 2:**
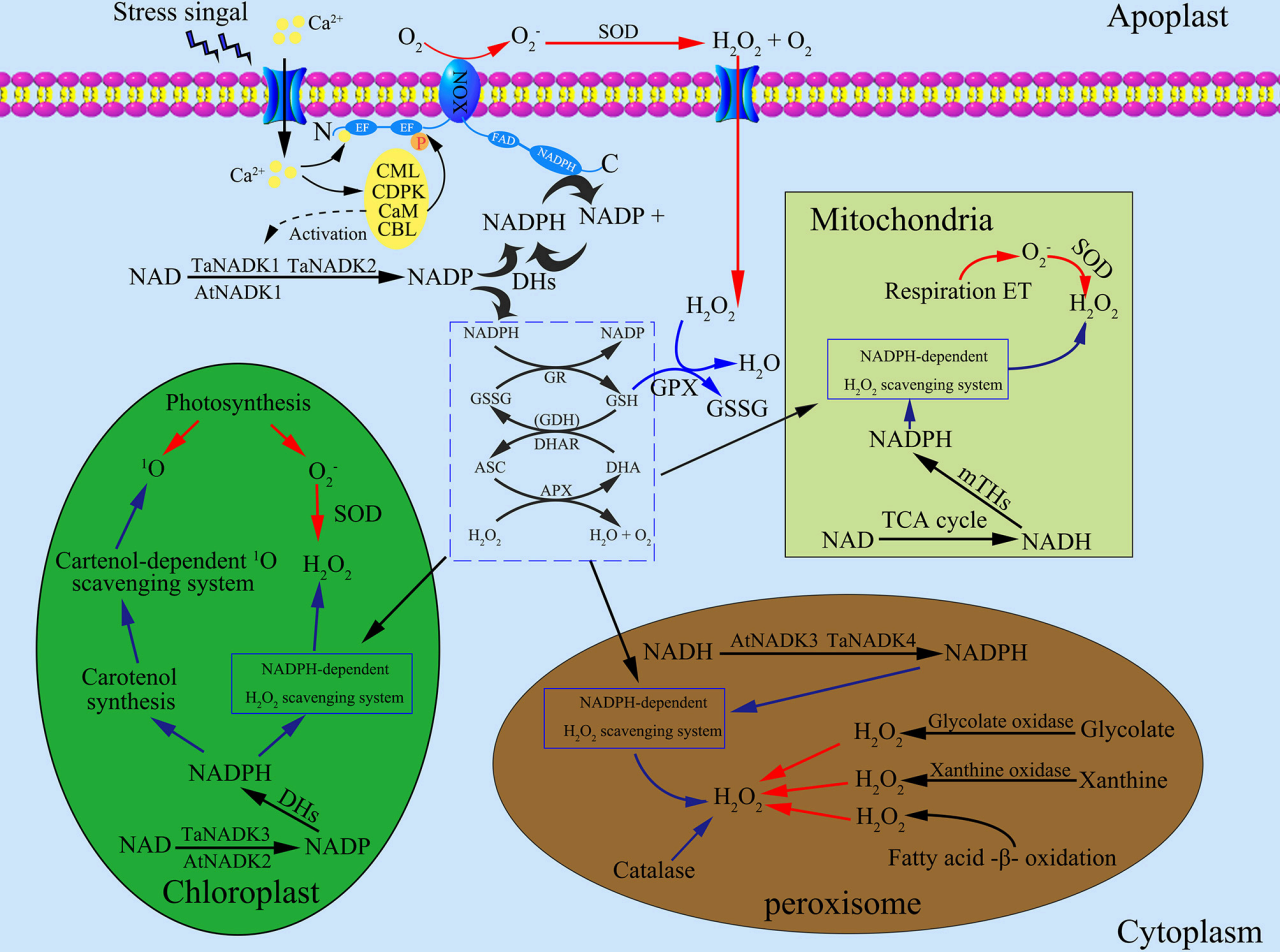
Roles of NADK proteins in maintaining reactive oxygen species (ROS) homeostasis in different subcellular compartments of plant cells. (1) The generation of ROS in cells. NADPH oxidases (NOXs) are located on the plasma membrane which can generate superoxide radical (${\text{O}}_{2}^{-}$) by catalyzing NADPH. Superoxide dismutase (SOD) can transform ${\text{O}}_{2}^{-}$ to oxygen and hydrogen peroxide (H_2_O_2_), a main kind of stable ROS. The complex of Ca^2+^/CaM participate in the production of ROS by activating NOXs. Under stress conditions, intracellular Ca^2+^ elevated according to Ca^2+^ influx from apoplast spaces, which leads to the activation of Ca^2+^ sensor proteins like the calcium-dependent protein kinase (CDPK), calmodulin (CaM), calmodulin-like protein (CML), and calcineurin B-like protein (CBL). CaMs also contribute to the activation of NADKs. In chloroplasts, photosynthesis is the main source of generating ROS: photosynthetic electron transport chain and Photosystem (PS) I or II release ${\text{O}}_{2}^{-}$, which is then transformed to H_2_O_2_ by SOD. Excited chlorophylls can also generate singlet oxygen (^1^O_2_), a kind of ROS with high energy. In mitochondria, ${\text{O}}_{2}^{-}$ is formed by the respiratory electron transport chain, which can be transformed to H_2_O_2_. In peroxisomes, H_2_O_2_ is generated through three main pathways including fatty acid oxidation, xanthine oxidation and glycolate oxidation. (2) The scavenging systems of ROS in cells. As an intermediate, ${\text{O}}_{2}^{-}$ can be immediately transformed to H_2_O_2_ by SOD, therefore, H_2_O_2_ becomes the main object of the ROS scavenging systems. The ascorbate-glutathione cycle and related enzymes including glutathione reductase (GR), dehydroascorbate reductase (DHAR), glutathione dehydrogenase (GDH) and ascorbate peroxidases (APX) function in both cytosol and other subcellular compartments such as in chloroplasts, mitochondria and peroxisomes. Besides, glutathione peroxidase (GPX) and catalase (CAT) can also detoxify H_2_O_2_. In the ROS scavenging systems, reducing power from the photosynthetic apparatus and NADPH are needed to support the operation. (3) NADKs distribute in different subcellular compartments, where they play an important role in the NADPH supply system. Firstly, NADKs can phosphorylate NAD to NADP in the different subcellular compartments. Then, dehydrogenases including glucose-6-phosphate dehydrogenase (G6PDH), 6-phosphogluconate dehydrogenase (6GPDH), NADP-dependent isocitrate dehydrogenase (ICDH), NADP-dependent malic enzyme (ME), NADP-dependent aldehyde dehydrogenase (ALDH) and NADP-dependent glutamate dehydrogenase (NADP–GDH) can transform NADP to NADPH. Transhydrogenases in mitochondria can also generate NADPH by transferring the hydrogen ion from NADH to NADP. Red arrows and blue arrows indicate the ROS generation and scavenging pathways, respectively. ASC, ascorbate; APX, ascorbate peroxidases; CAT, catalase; DHA, dehydroascorbate; DHAR, dehydroascorbate reductase; GR, glutathione reductase; GSH, glutathione; GSSG, oxidized glutathione; GDH, glutathione dehydrogenase; ROS, reactive oxygen species.

The multiple subcellular localizations of NADKs may have an important biological significance in plant cells. As shown in Figure 2, NADKs not only participate in NADP-dependent metabolism in different organelles, but also control the NAD(H)/NADP(H) balance and ROS homeostasis in the different subcelluar compartments. As is well known, the three organelles, chloroplasts, mitochondria and peroxisomes, are the major source of ROS in plants (Neill et al., 2002). Therefore, at first, the multiple and compartmental localization of NADKs could be efficient for the control of ROS homeostasis in these organelles. Secondly, this compartment localization can confer more diverse functions to NADKs. For example, the chloroplast-located NADK (AtNADK2) also functions in photosynthetic efficiency, xanthophyll cycle and substrate production (Takahashi et al., 2006; Takahara et al., 2010; Onda et al., 2014). In addition, the peroxisome-located NADK (AtNADK3) also might function in fatty acid β-oxidation (Waller et al., 2010). The NADHK (POS5) from *S. cerevisiae* is also involved in fatty acid β-oxidation in peroxisomes by supplying NADPH (Shi and Li, 2006). All these results indicate that different subcellular distribution of plant NADKs is essential for plant growth and development.

## Differential expression patterns of plant NADK genes

As reported previously, we identified 74 NADK genes from 24 species representing the eight major plant lineages within the supergroup Plantae: glaucophytes, rhodophytes, chlorophytes, bryophytes, lycophytes, gymnosperms, monocots, and eudicots (Li et al., 2014). Then, we found 11 genes in the wheat genome encoding the NADK isoforms (Wang et al., 2016). These NADK genes exhibited differential expression patterns in different tissues of plants at different stages during plant development and growth. In bean, NADK activity was found to be essential for early growth metabolism of the plant (Stephan and Laval-Martin, 2000). The expression of NADK genes also shows obvious tissue-specificity. In *Arabidopsis thaliana*, the three NADK genes, *AtNADK1, AtNADK2*, and *AtNADK3*, were strong tissue-specifically expressed where *AtNADK1* was expressed mainly in roots, *AtNADK2* mainly in leaf tissue and *AtNADK3* strongly in vasculature tissue even though all *AtNADKs* exhibited same expression level in reproductive tissues (Waller et al., 2010). NADK genes in rice and wheat were also tissue-specifically expressed during vegetative and reproductive developmental stages. For example, *TaNADK1* was strongly expressed in root during germination, *TaNADK2* was highly expressed in pistil, caryopsis and endosperm during the reproductive stage, whereas *TaNADK3* and *TaNADK4* were highly expressed in floral bracts and embryo, respectively (Li et al., 2014; Wang et al., 2016).

**Figure 3:**
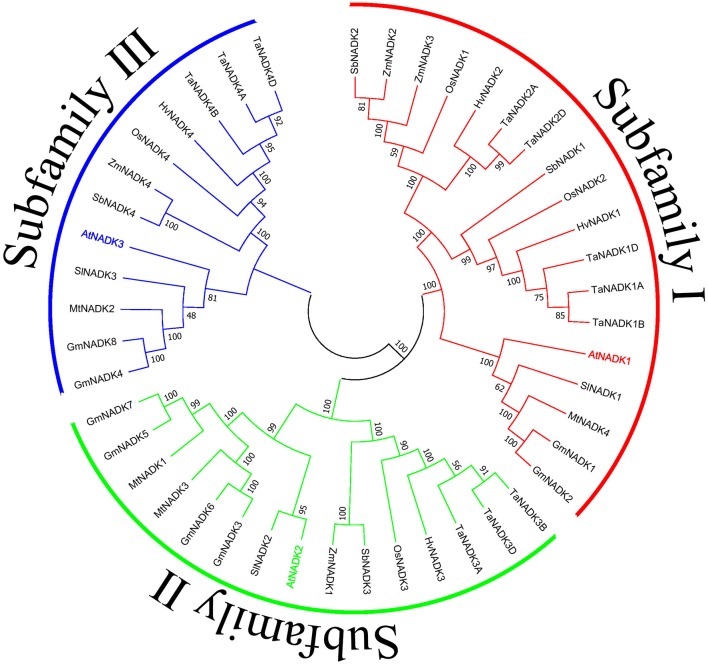
Phylogenetic relationship of nine plant NADK families. The tree was constructed using the Neighbor-Joining method with the full length amino acid sequences of the NADKs from nine plant species. Numbers above the nodes represent bootstrap values from 1,000 replications. Subfamilies were divided as previously reported (Li et al., 2014).

To get further insights on NADK genes during growth and development of plants, we investigated the developmental and anatomical expression profiles of NADK genes from nine plant species based on the microarray and mRNA sequencing data from Genevestigator (https://genevestigator.com/gv/) (Table 2, Figures S1, S2). A phylogenetic tree was also made to illustrate the phylogenetic relationship between these NADK families (Figure 3). As can be seen, NADK gene families from nine plant species, including *AtNADK*s in *Arabidopsis thaliana, SlNADK*s in *Solanum lycopersicum, GmNADK*s in *Glycine max, MtNADK*s in *Medicago truncatula, OsNADK*s in *Oryza sativa, HvNADK*s in *Hordeum vulgare, ZmNADK*s in *Zea mays, SbNADK*s in *Sorghum bicolor* and *TaNADK*s in *Triticum aestivum*, are expressed at almost all developmental stages, even though some members are barely expressed at some developmental stages. For example, *AtNADK3* is barely expressed at the bolting stage and mature siliques stage, and *HvNADK1* shows no expression at anthesis stage. Due to *Triticum aestivum* being an allohexaploid plant, a total of 11 NADK genes were identified encoding the four NADK isoforms in this species (Wang et al., 2016)*. TaNADK1A, TaNADK1B*, and *TaNADK1D* are distributed in different subchromosomes which all encode TaNADK1. As shown in Figure S1, although the genes encoding the same NADK isoform in wheat have similar expression patterns in different developmental stages, their paralogous genes have different expression patterns. For example, *TaNADK1A, B* and *D* were strongly expressed in booting stage whereas *TaNADK4A, B* and *D* were highly expressed at ripening stage. The expression patterns of these NADK families also show some tissue-specificity, even though most genes are expressed almost throughout the whole plant (Figure S2). Tissue-specific and/or developmental stage-dependent expression implies diversity in functions of NADK genes in plant growth and development. Although the exact meanings of the specific expression for the NADKs have not been well studied in plants, the results obtained here demonstrate that the differential expression patterns of these NADKs may be related to their functions. For instance, NADKs from subfamily II such as AtNADK2, and TaNADK3 have higher expression levels in leaves (Table 2), due to their localization in chloroplasts of the plants (Chai et al., 2005; Wang et al., 2016).

**Table 2:** Developmental and anatomical expression patterns of NADK family genes in plants.

**Species**	**Gene name**	**Accession No**.	**Germination**	**Seedling**	**Root**	**Leave**	**Stem**	**Flower**	**Inflorescence**	**Seed**
*Arabidopsis thaliana*	*AtNADK1*	AT3G21070.1	•	•	••	•	•	•	•	••
	*AtNADK2*	AT1G21640.2	•	••	••	•••	•••	••	••	•
	*AtNADK3*	AT1G78590.1	•	•	••	•	•	••	••	••
*Solanum lycopersicum*	*SlNADK1*	Solyc01g008560.2	NA	•	••	•	•	••	NA	•
	*SlNADK2*	Solyc11g005100.1	NA	NA	NA	NA	NA	NA	NA	NA
	*SlNADK3*	Solyc07g062150.2	NA	NA	NA	NA	NA	NA	NA	NA
*Glycine max*	*GmNADK1*	Glyma.11G093600	•••	••	••	••	NA	•	•	•
	*GmNADK2*	Glyma.12G020100	••	•••	•••	••	NA	••	••	•
	*GmNADK3*	Glyma.06G117300	•••	•	•	•	NA	•	•	••
	*GmNADK4*	Glyma.12G234000	••	••	••	•	NA	•	•	•
	*GmNADK5*	Glyma.13G032400	•	•••	•	••	NA	•	••	•
	*GmNADK6*	Glyma.04G245800	••	•	••	••	NA	•	•	••
	*GmNADK7*	Glyma.14G154600	•	••	•	••	NA	•	•	•
	*GmNADK8*	Glyma.13G264900	••	••	••	••	NA	••	•	•
*Medicago truncatula*	*MtNADK1*	MTR_1g029620	•	•	•	•••	••	••	••	•
	*MtNADK2*	MTR_2g073350	•	••	•	•	••	••	••	••
	*MtNADK3*	MTR_3g088575	••	••	•	••	••	••	•••	••
	*MtNADK4*	MTR_4g076990	••	••	••	•••	•	••	•	•
*Oryza sativa*	*OsNADK1*	LOC_Os01g72690.1	••	••	••	•	••	•	•••	••
	*OsNADK2*	LOC_Os05g32210.1	••	••	•	•	••	••	••	•
	*OsNADK3*	LOC_Os11g08670.1	•	•	•	••	••	•	•	••
	*OsNADK4*	LOC_Os09g17680.1	••	•	•	•	••	•	••	•
*Hordeum vulgare*	*HvNADK1*	HORVU1Hr1G056560	•	•	••	•	•	•	•	•
	*HvNADK2*	HORVU3Hr1G109740	NA	NA	NA	NA	NA	NA	NA	NA
	*HvNADK3*	HORVU4Hr1G017680	NA	NA	NA	NA	NA	NA	NA	NA
	*HvNADK4*	HORVU5Hr1G056620	•	•	•	•	•	•	•	•
*Zea mays*	*ZmNADK1*	GRMZM2G046498_T02	•	•	•	•	••	•	••	•
	*ZmNADK2*	GRMZM2G059073_T02	•	•	••	••	••	••	••	•
	*ZmNADK3*	GRMZM2G138342_T01	••	••	••	••	••	••	•••	•
	*ZmNADK4*	GRMZM2G006678_T01	•	•	•	•	•	•••	••	•••
*Sorghum bicolor*	*SbNADK1*	SORBI_009G123700	NA	••	••	•	•	•	•	•
	*SbNADK2*	SORBI_003G432100	NA	••	•	•	••	••	•••	••
	*SbNADK3*	SORBI_005G068700	NA	••	•	••	••	•	•	•
	*SbNADK4*	SORBI_002G176800	NA	•	•	•	•	•	•	••
*Triticum aestivum*	*TaNADK1A*	Traes_1AL_EEFF7B5F8	••	••	•	•	••	••	••	•
	*TaNADK1B*	Traes_1BL_735ECE522	•	••	•	•	••	•	•	•
	*TaNADK1D*	Traes_1DL_8F5175629	•	••	•	•	••	•	•	•
	*TaNADK2A*	Traes_3AL_2DFA99E9A	•	•••	•••	•	••	••	••	•
	*TaNADK2D*	Traes_3DL_AF4B6651B	••	•••	•••	•	••	••	••	•
	*TaNADK3A*	Traes_4AL_8758B00CD	•	••	•	••	•	•	•	•
	*TaNADK3B*	Traes_4BS_5353939DF	••	••	•	••	•	••	•	•
	*TaNADK3D*	Traes_4DS_01D383F11	••	••	•	••	•	•	•	•
	*TaNADK4A*	Traes_5AL_1B961C5C0	•	••	•	•	•	•	•	••
	*TaNADK4B*	Traes_5BL_26C8767EF	•	••	•	•	•	•	•	••
	*TaNADK4D*	Traes_5DL_348B0D6E3	•	••	•	•	•	•	•	••

*Summary of relative expression patterns of NADK genes in nine plant species, including Arabidopsis thaliana, Solanum lycopersicum, Glycine max, Medicago truncatula, Oryza sativa, Hordeum vulgare, Zea mays, Sorghum bicolor and Triticum aestivum. Expression profiles of nine NADK family genes in different developmental stages and tissues were obtained from the microarray and mRNA sequencing data in Genevestigator (Figures S1, S2). •, weak expression; ••, moderate expression; •••, strong expression; NA, not available*.

## Vital roles of NADKs in NADPH dependent metabolic pathways

### Responsible for NADPH production

NADPH is an essential cofactor used in anabolic reactions, such as lipid and nucleic acid syntheses, which require NADPH as reducing agent. The source of NADPH biosynthesis consists of a NADK-mediated direct biosynthesis pathway and a series of dehydrogenase-mediated pathways in cells. In many studies, it was shown that after the phosphorylation of NAD by NADK, the resulting NADP could be re-reduced immediately into NADPH. In addition, NADH kinase can catalyze NADH to synthesize NADPH directly. POS5 in *S. cerevisiae* and AtNADK3 in *Arabidopsis thaliana* are representatives of NADH kinases which have been well studied (Outten and Culotta, 2003; Turner et al., 2005). These two NADKs have NADH kinase activity much higher than NAD kinase activity. NADH kinase constructs the most efficacious pathway to produce NADPH.

**Figure 4:**
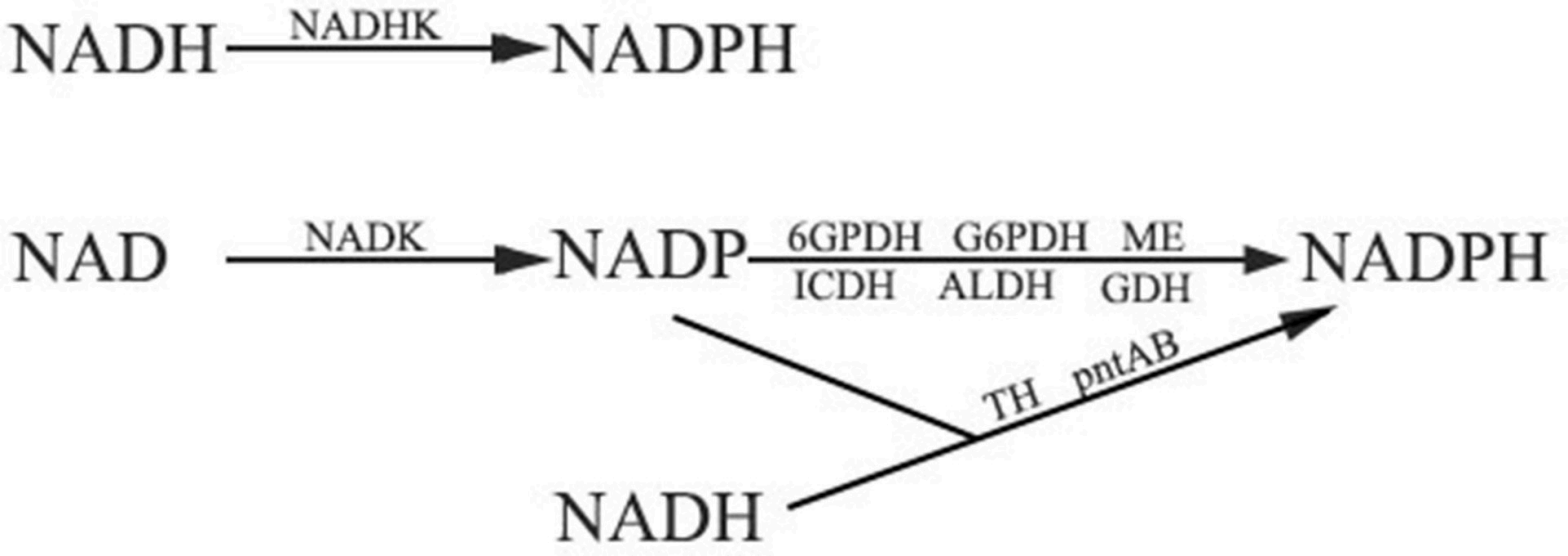
The pathways of NADPH production. NAD(H) kinases play an important role in both the two NADPH generation pathways. Firstly, NADH kinase (NADHK) can catalyze NADH to synthesize NADPH directly. Secondly, NADPH can be generated by glucose-6-phosphate dehydrogenase (G6PDH), 6-phosphogluconate dehydrogenase (6GPDH), NADP-dependent isocitrate dehydrogenase (ICDH), NADP-dependent malic enzymes (ME), NADP-dependent aldehyde dehydrogenase (ALDH), and NADP-dependent glutamate dehydrogenase (GDH). Transhydrogenase (TH) and proton-trans-locating transhydrogenase (pntAB) can also generate NADPH. NAD kinase functions in providing NADP to these enzymes.

The other pathway for NADPH production depends on the function of a series of dehydrogenases (DHs) and transhydrogenases (THs), but all of the enzymes need NADP produced by NAD kinase. NADP-dependent dehydrogenase can reduce NADP to NADPH and NADH-dependent transhydrogenases can catalyze the transfer of proton from NADH to NADP. Glucose-6-phosphate dehydrogenase (G6PDH) and 6-phosphogluconate dehydrogenase (6GPDH) are two enzymes that participate in the pentose phosphate pathway. G6PDH is a cytosolic enzyme which can reduce NADP to NADPH while oxidizing glucose 6-phosphate (Kletzien et al., 1994). 6GPDH also can catalyze NADP to synthesize NADPH (Rosemeyer, 1987). NADP-dependent isocitrate dehydrogenase (ICDH) is a key enzyme in the citric acid cycle (Krebs cycle) which uses NADP as a cofactor to synthesize NADPH. It is localized to the cytosol as well as to the mitochondrion and the peroxisome (Ying, 2008). In addition, other NADP dependent enzymes including NADP-dependent malic enzyme (ME), aldehyde dehydrogenase (ALDH) family Ald6p, NADP-dependent glutamate dehydrogenase (NADP-GDH), and mitochondrial transhydrogenase (mTH) and NADPH:NAD oxidoreductase (Si-specific) (pntAB), which are located in the cytosol or the mitochondria, can also contribute to NADPH production (Grabowska and Chelstowska, 2003; Sauer et al., 2004; Rydström, 2006; Singh et al., 2007). The main system of NADPH generation in plants has been summarized in Figure 4, emphasizing the fundamental role of NADKs in NADPH production.

### Functioning in photosynthesis and other metabolic reactions

As described above, there are three NADKs existing in *Arabidopsis thaliana* but only AtNADK2 is located in chloroplasts. In chloroplasts, NADP as the production of NADK accepts the electrons at the last step of the light dependent reactions in photosynthesis and its reduced form NADPH also participates in carbon fixation in light independent reactions, which indicates the importance of AtNADK2 in photosynthesis. In addition, as the major enzyme for NADPH production in chloroplasts, the functions of AtNADK2 in different metabolism pathways have been well disclosed in recent years. It was found that an *AtNADK2* knockout mutant (*atnadk2*) showed growth inhibition and smaller rosette leaves with a pale yellow color due to the reduced chlorophyll content (Chai et al., 2005). In addition, the mutant can accumulate much more Pchlide (the precursor of chlorophyll) and Mg-Protoporphyrin IX than the wild type (Chai et al., 2005). Because the conversion of Pchlide to Chlide needs NADPH as reducing energy, due to the lack of *AtNADK2* expression, the contents of NADP and NADPH were obviously reduced in the mutant and thus affected chlorophyll biosynthesis (Chai et al., 2005). Moreover, the efficiency of photosynthetic electron transport and the quantum yield of open reaction centers of Photosystem II were found to be decreased in the mutant but the non-photochemical quenching attributed to energy dissipation from the xanthophyll cycle increased (Takahashi et al., 2006), suggesting the essential role of AtNADK2 for the proper photosynthetic machinery of Photosystem II and the xanthophyll cycle. On the other hand, overexpression of *AtNADK2* led to higher Calvin-Bensen cycle activity, Rubisco activity, NADP(H) concentration, nitrogen assimilation, as well as glutamine and glutamate and some other amino acids production in the transgenic plants (Takahashi et al., 2009).

As the regulator of co-enzyme homeostasis, NADK also functions in many other biosynthesis reactions. Although NADK directly contributes to NADP production in cells, it seems that the concentration of NADPH is always affected. In the *atnadk2* mutant, the concentration of NADPH is lower compared to the wild type (Chai et al., 2005; Takahashi et al., 2006). Since NADPH can be utilized as reducing power in many anabolic pathways and overexpression of NADK can improve NADPH concentration efficiently, overexpressing NADK becomes a smart strategy to improve the products in anabolic pathways. For instance, overexpression of *AtNADK2* in rice led to the increase in the levels of amino acids and several sugar phosphates including ribose 1,5-bisphosphate compared with the wild type (Takahara et al., 2010). The transgenic lines also showed higher electron transport and carbon dioxide (CO_2_) assimilation rates (Takahara et al., 2010) and nitrogen-rich amino acids biosynthesis under elevated CO_2_ (Onda et al., 2014), implying the essential roles of NADKs in plant cell metabolism.

Evidences from microbes also proved that the yields of a series of products of interest can be elevated by overexpressing NADKs. Poly(3-hydroxybutyrate) is a kind of intracellular insoluble material forming granules in bacteria, which has excellent physical and chemical properties and has a considerable prospect in new material development. It was reported that overexpressing NADK in recombinant *E. coli* increased the accumulation of poly(3-hydroxybutyrate) (Li et al., 2009; Zhang et al., 2015). This method was also utilized to improve the yield of a series of chemical products like L-ornithine and L-isoleucine (Shi et al., 2012; Jiang et al., 2013).

In addition, overexpression of NADH kinase can also increase the yields of carbohydrates and carotenoids by improving NADPH concentration directly. Lee et al. (2013) found that the yields of guanosine diphosphate (GDP)-L-fucose and ε-caprolactone were significantly improved by overexpressing NADH kinase POS5p of *S. cerevisiae* in *E. coli*. A similar result could be also obtained by improving carotenoid production. Glucose-6-phosphate dehydrogenase (Zwf1) and the NADH kinase Pos5 are the two main NADPH-supplying sources in *S. Cerevisiae*, overexpression of Pos5 led to 85 and 39% of lycopene and β-carotene increases in the recombinant *S. cerevisiae*, respectively, as compared to the Zwf1 overexpressed strain. Overexpressing Pos5 is more effective than overexpressing Zwf1 for the production of carotenoids (Zhao et al., 2015).

For further improving the yield of products of interest, a more comprehensive and valid method has been adopted. Since both NADK and NADP-dependent dehydrogenases can improve the concentration of NADPH, overexpression of the two kinds of enzymes simultaneously should be expected to results in elevated NADPH production. In an engineered shikimic acid-producing *E. coli* strain, it was reported that the increasing NADPH availability, achieved by overexpressing *pntAB* (a transhydrogenase gene) and/or *NADK* genes, significantly enhanced shikimic acid production (Cui et al., 2014). Isobutanol is an alternative biofuel. Shi et al. (2013) found that overexpressing both transhydrogenase (*pntAB*) and NAD kinase (*yfjB*) genes significantly increased isobutanol production in recombinant *E. coli*. Using the same strategy can also promote the production of free fatty acids (Wu et al., 2014).

## Acting as the key players for the response of higher plants to various environmental stresses

### Responding to abiotic and biotic stresses in different plants

Improving the level of the reducing power is an efficient strategy to resist the oxidative stresses provoked by environmental stimulation, which results in NADK having an indispensable role in responding to environmental stresses. NADK activity of plants has variations under different environmental stresses. In wheat, NADK activity was decreased in leaves by water-deficit stress (Zagdanska, 1990) but increased in apical segments of roots after exposure to aluminum (Slaski, 1995). When treated by indoleacetic acid (IAA), its activity in wheat coleoptile was up-regulated (Li et al., 1999). Under conditions of cold-shock stress, the Ca^2+^-dependent NADK activity in green bean leaves was raised (Ruiz et al., 2002). In strawberry, Gu (2007) found that ethylene can down-regulate the activity of NADK, reducing the concentration of NADPH, which accelerates the ripening of fruit indirectly. But under cold environmental conditions, the activity was raised, leading to an increase in NADPH concentration to prevent the fruits from ripening (Gu et al., 2007).

In addition, many studies have shown that the expression of NADK family genes can be strongly stimulated by a number of environmental stresses in plants. The expression of *A. thaliana AtNADK1* was up-regulated 3–8-fold under several treatments including ionizing radiation, hydrogen peroxide (H_2_O_2_) application and plant pathogen interactions (Berrin et al., 2005). *AtNADK3* was also inducible by environmental treatments, its expression was up-regulated more than 5-fold by methyl viologen (MV) treatment and raised 3-fold by NaCl treatment (Chai et al., 2006). When treated with mannitol or low temperature, it had 6-fold and 8-fold up-regulation, respectively (Chai et al., 2006). Although there are some species specificities between the orthologous and/or paralogous genes of the cereal NADK genes, their transcripts in wheat, rice and maize were all strongly affected by one or several environmental stress treatments such as cold, heat, dehydration (PEG), salt (NaCl), oxidative stress (MV), and hormones including abscisic acid (ABA), methyl jasmonic acid (MeJA), and salicylic acid (SA) (Wang et al., 2016), implying their crucial roles in the abiotic stress response and hormone signaling regulation.

To better understand the functions of NADK family genes in plant stress responses, the inducible expression patterns of NADK family genes from eight kinds of plants have been summarized here based on the microarray and mRNA sequencing data from Genevestigator (Table 3, Figure S3). The results show that the expression of NADK family genes was not only induced by abiotic stresses and hormone treatments, but also induced by biotic stresses. For instance, *TaNADK1*s, *TaNADK3*s, and *TaNADK4*s were down-regulated but *TaNADK2*s were up-regulated under *F. graminearum* attack. *TaNADK2*s were also up-regulated under *P. graminis* attack. According to the expression profiles of eight kinds of NADK family genes including *AtNADKs, OsNADKs, TaNADKs, ZmNADKs, HvNADKs, MtNADKs, GmNADKs*, and *SlNADKs*, NADK family genes from different species were induced to varying degrees by abiotic/biotic stresses and hormone treatments, exhibiting high diversity in response to various environmental stresses between the orthologous and/or paralogous genes (Table 3, Figure S3). Besides, the expression level of a particular NADK has a different response to different concentrations of a certain treatment. For example, *AtNADK3* transcripts were up-regulated more than 5-fold after a 6 h treatment with 1 μM MV, but they were down-regulated deeply after a 12 h treatment with 30 μM MV, implying its spatiotemporal expression character. Although we could not exactly distinguish the certain roles of each NADK gene in plant stress tolerance at the current stage, all of these results underscore the functional diversity and divergence of NADK family genes of plants in responding to a number of abiotic and biotic stresses.

**Table 3:** Inducible expression patterns of NADK family genes in eight plant species under drought, salt, heat, and cold stresses.

**Species**	**Gene name**	**Accession No**.	**Drought**	**Salt**	**Heat**	**Cold**
*Arabidopsis thaliana*	*AtNADK1*	AT3G21070.1	•••	•••	•	•
	*AtNADK2*	AT1G21640.2	°	•	••	••
*Solanum lycopersicum*	*SlNADK1*	Solyc01g008560.2	••	••	••	NA
*Glycine max*	*GmNADK1*	Glyma.11G093600	°	NA	•	NA
	*GmNADK2*	Glyma.12G020100	•	NA	••	NA
	*GmNADK3*	Glyma.06G117300	••	NA	•	NA
	*GmNADK4*	Glyma.12G234000	••	NA	••	NA
	*GmNADK5*	Glyma.13G032400	••	NA	°	NA
*Medicago truncatula*	*MtNADK1*	MTR_1g029620	•••	•••	NA	NA
	*MtNADK2*	MTR_2g073350	••	••	NA	NA
	*MtNADK3*	MTR_3g088575	••	°	NA	NA
	*MtNADK4*	MTR_4g076990	••	••	NA	NA
*Oryza sativa*	*OsNADK1*	LOC_Os01g72690.1	•	°	••	•••
	*OsNADK2*	LOC_Os05g32210.1	•	•	•	••
	*OsNADK3*	LOC_Os11g08670.1	••	•	•••	••
	*OsNADK4*	LOC_Os09g17680.1	••	•	••	°
*Hordeum vulgare*	*HvNADK1*	HORVU1Hr1G056560	NA	•	°	••
	*HvNADK4*	HORVU5Hr1G056620	NA	°	•	•
*Zea mays*	*ZmNADK1*	GRMZM2G046498_T02	••	NA	••	•••
	*ZmNADK2*	GRMZM2G059073_T02	•	NA	••	••
	*ZmNADK3*	GRMZM2G138342_T01	•	NA	••	•
	*ZmNADK4*	GRMZM2G006678_T01	••	NA	••	•
*Triticum aestivum*	*TaNADK1A*	Traes_1AL_EEFF7B5F8	•	NA	••	•
	*TaNADK1B*	Traes_1BL_735ECE522	••	°	•	••
	*TaNADK1D*	Traes_1DL_8F5175629	°	NA	°	•
	*TaNADK2A*	Traes_3AL_2DFA99E9A	••	NA	•	••
	*TaNADK2D*	Traes_3DL_AF4B6651B	••	NA	•	••
	*TaNADK3A*	Traes_4AL_8758B00CD	••	•	••	••
	*TaNADK3B*	Traes_4BS_5353939DF	••	NA	••	•
	*TaNADK3D*	Traes_4DS_01D383F11	•••	NA	••	°
	*TaNADK4A*	Traes_5AL_1B961C5C0	•	NA	•	•
	*TaNADK4B*	Traes_5BL_26C8767EF	••	NA	•	•
	*TaNADK4D*	Traes_5DL_348B0D6E3	•	NA	°	••

*Summary of relative expression level of NADK family genes in eight plant species, including Arabidopsis thaliana, Solanum lycopersicum, Glycine max, Medicago truncatula, Oryza sativa, Hordeum vulgare, Zea mays, and Triticum aestivum. The inducible expression profiles of eight NADK family genes under drought, salt, heat and cold stresses were inferred from the microarray and mRNA sequencing data reported by Genevestigator (Figure S3). The numbers of the dot indicated the expression levels compared with wild type (from low to high). Green dot and red dot means the down-regulation and up-regulation, respectively. Hollow dot (°) means the expression level barely changed compared with the controls*.

### Participating in ROS homeostasis as the NADPH supplier in plant cells

During plant growth, ROS are produced as metabolic by-products. However, when plants experience different kinds of environmental stresses including abiotic stresses like cold, heat, salt, drought, and also biotic stresses including different pathogen attacks, ROS are produced as signaling components in stress tolerance (Neill et al., 2002; Edreva, 2005; Foyer and Noctor, 2005; Noctor and Foyer, 2016). Although low levels of ROS play an important role in signaling events, excessive ROS are harmful for plants. They can lead to cell death by oxidative processes including membrane lipid peroxidation, protein oxidation, enzyme inhibition, and DNA and RNA damage (Mittler, 2002; Circu and Aw, 2010; Liu and He, 2016). The excess ROS induced by environmental stresses must be scavenged. Therefore, ROS homeostasis is very important for the survival of plants under adverse environmental conditions.

To demonstrate the importance of NADK in ROS scavenging systems, we summarized here the main sources of ROS producing systems, anti-oxidative systems and NADPH supplying systems in different subcellular compartments of plants (Figure 2). In the cytosol, the plasma membrane-targeted NADPH oxidases (NOXs) can produce ROS by utilizing intracellular NADPH as reductant (Bedard et al., 2007). Chloroplasts and mitochondria are not only powerhouses, but also the main ROS producers in plant cells because of photosynthetic and respiratory electron transfer chains, respectively (Asada and Takahashi, 1987; Asada, 1999; Maxwell et al., 1999; Dat et al., 2000). Excited chlorophyll can also generate singlet oxygen (^1^O_2_), a high-energy form of oxygen (Asada and Takahashi, 1987). In peroxisomes, ROS can be generated from several ways including glycolate oxidation, fatty acid β-oxidation, and xanthine oxidation (Corpas et al., 2001). On the other hand, the ascorbate-glutathione cycle (Halliwell-Asada cycle) and related enzymes including glutathione reductase (GR), dehydroascorbate reductase (DHAR), glutathione dehydrogenase (GDH) and ascorbate peroxidases (APX) play crucial roles in the scavenge of ROS induced by environmental stresses (Noctor and Foyer, 1998; Horemans et al., 2000; Foyer et al., 2006). Besides, carotenoids and catalase also can scavenge ROS efficiently in chloroplasts and peroxisomes, respectively (Asada and Takahashi, 1987; Willekens et al., 1997). As the only known enzyme to synthesize NADP(H), NADK is indispensable in anti-oxidative stress systems because NADP(H) can provide the main reduction power for the ROS scavenging system (Mittler, 2002) and therefore NADK plays crucial roles in ROS homeostasis of plant cells. Ca^2+^/CaM also played a dual role in NOX-mediated ROS production and NADPH dependent scavenging (Figure 2). The activation of NOX needs Ca^2+^ binding to its EF-hand motif and the phosphorylation by Ca^2+^ dependent protein kinase, such as calcium-dependent protein kinase (CDPK), calmodulin (CaM), and calmodulin-like protein (CML) or calcineurin B-like protein (CBL) (Kobayashi et al., 2007; Ogasawara et al., 2008; Drerup et al., 2013; Dubiella et al., 2013). Roberts and Harmon (1992) reviewed how CaMs contribute to Ca^2+^/CaM dependent NADK activation in higher plants. This further emphasizes the significance of NADK in ROS homeostasis.

As described above, NADK isoforms have multiple subcellular localizations, which can supply sufficient NADPH in different subcellular compartments in plants. In chloroplasts, NADPH not only participates in NADPH-dependent anti-oxidative systems directly, but also tightly relates to carotenoids biosynthesis (Hornero-Méndez and Britton, 2002). As a chloroplast-targeted NADK in *Arabidopsis thaliana*, AtNADK2 is essential in maintaining the NADPH levels of chloroplasts. Takahashi et al. (2006) found that the *atnadk2* mutant showed an aberrant de-epoxidation state of its xanthophyll cycle carotenoids which may relate to the pale green phenotype and growth inhibition of the mutant plants. In cyanobacteria, MV treatment can damage macromolecules and membranes by accepting electron from Photosystem I and reducing O_2_ to ${\text{O}}_{2}^{-}$. It was reported that the *sll1415* mutant (a kind of NADK knockout mutant) exhibited enhanced sensitivity to MV treatments, indicating that the NADK is essential to resist oxidative stress (Gao and Xu, 2012). In peroxisomes, NADK (like AtNADK3 and TaNADK4) was suggested not only to participate in the balancing of NAD(H) and NADP(H) but also to function in fatty acid β-oxidation (Waller et al., 2010; Wang et al., 2016). All these results suggest that NADK plays a crucial role in ROS homeostasis in different subcellular compartments of plants. By improving the NADPH level efficiently, NADK functions in both ROS generation and scavenging through NADPH-dependent NOXs and NADPH-dependent scavenging systems, respectively.

### Contributing to intracellular redox balance

NADPH/NADP, AsA (ascorbate)/DHA (dehydroascorbate) and GSH (glutathione)/GSSG (oxidized glutathione) are the three major intracellular redox couples which are widely involved in metabolism, signal transduction and transcriptional regulation of plants (May et al., 1998; Pollak et al., 2007a; Bilan et al., 2015). Due to the vital roles of NADK in the conversion of NAD to NADP and the synthesis of NADPH, it firstly contributes to the balance of NAD(H) and NADP(H) in cells. It was reported that the metabolic redox balance between NADH and NADPH is essential for cell survival under oxidative stress and the increase in NADPH by NADK is needed by cells to protect against oxidative challenges in *Pseudomonas fluorescens* (Mailloux et al., 2011). Gray et al. (2012) found that NADK regulates the ratio of NADPH/NADP and affects the intracellular redox and oxidative defense in the pancreatic β-cells. In *Arabidopsis thaliana*, Berrin et al. (2005) found that AtNADK1 may contribute to the maintenance of redox status in cells to withstand gamma irradiation and MV-induced oxidative stress. AtNADK2 also functions in the balance of NAD(H) and NADP(H) to cope with environmental stresses in the plant. It was found that, the content of NAD(H) was increased but that of NADP(H) was decreased in the *AtNADK2* deletion mutant *atnadk2*: consequently, the tolerance of the mutant to various environmental stresses such as UVB, drought, heat and salt was markedly reduced (Chai et al., 2005; Takahashi et al., 2006, 2009; Takahara et al., 2010). In addition, previous studies showed that *A. thaliana* AtNADK3 is a peroxisomal enzyme (Waller et al., 2010) and preferentially phosphorylates NADH over NAD to yield NADPH in the plant (Turner et al., 2004, 2005). More recently, we found that lack of OsNADK1, a cytosolic NADK in rice, significantly increased the ratio of NAD(H)/NADP(H) but decreased the ratio of NADPH/NADP, implying an important role of OsNADK1 in the balance of NAD(H) and NADP(H) of rice (data not shown).

As the two other intracellular redox couples, roles of AsA/DHA and GSH/GSSG in plant stress responses or tolerance were paid great attention in the past decades (May et al., 1998). Li and van Staden (1998) found that the content of AsA was decreased in maize under drought treatment. However, Schwanz et al. (1996) reported that the total AsA was decreased but the reduction AsA was increased in oak trees under drought conditions. In the roots of tomato, Shalata et al. (2001) found that the content of AsA was decreased but DHA increased, leading to the ratio of AsA/DHA in a salt sensitive cultivar being decreased under salt stress conditions. In other reports, it was shown that GSH/GSSG ratios might affect the whole redox potential of cells by influencing NAD/NADP metabolism. Decreased GSH/GSSG ratios lead to decreased NADPH/NADP ratios, and the decreased NADPH/NADP ratios may affect NADH/NAD ratios due to the NADKs modulating capacity (Ying, 2008). In fact, a knockout mutant of *A. thaliana AtNADK3, atnadk3*, showed a decrease of NADPH, and also exhibited a decreased ratio of GSH/GSSG (Chai et al., 2006). In our study, a marked decrease in the ratios of AsA/DHA and GSH/GSSG was also observed in a rice *OsNADK1*-knockout mutant (data not shown). All these results strongly suggest that NADK plays crucial roles in the maintaining intracellular redox balance of plants.

### Conferring to stress tolerance

Several reports have illustrated that NADK deficient mutants become more sensitive to environmental stresses in *Arabidopsis thaliana*. When treated by MV a potent oxidant, *AtNADK1*-deficient mutant plants can produce a high level of anthocyanin, a regulator when plants are subjected to oxidative stress, indicating that the *atnadk1* mutant is more sensitive to oxidative stress (Berrin et al., 2005). In an *AtNADK2-*deficient mutant, the total NADPH level is lower than wild type, which also displayed hypersensitivity to oxidative stress induced by UVB, drought, heat shock or salinity (Chai et al., 2005). The *AtNADK3*-deficient mutant also showed hypersensitivity to oxidative stress in both seed germination and seedling growth (Chai et al., 2006). In contrast, overexpressing NADK confers tolerance to oxidative stress. In rice overexpressing *AtNADK2* transgenic lines, the ratio of GSH/GSSG was significantly elevated, which led to a slower decrease in the photochemical efficiency of Photosystem II and total chlorophyll, and a lower concentration of malondialdehyde, a marker of lipid peroxidation, indicating a higher stress tolerance of the transgenic plants (Takahara et al., 2010). Petriacq et al. (2012) also proved that higher NADPH levels can enhance the tolerance of *Arabidopsis thaliana* transgenic plants to the pathogen *Pseudomonas syringae* pv. *tomato Pst-AvrRpm1*. Recently, we found that overexpression of *OsNADK1* in rice led to high tolerance of the plant to drought stress. The transgenic plants exhibited a high content of proline and transcripts of several redox-sensitive genes such as *OsDREB1B*, whereas the loss of function plants of *OsNADK1* are opposite in this regard (data not shown). Overexpression of wheat *TaNADK4* in rice also confers to the plant a strong tolerance to drought (data not shown).

Interestingly, due to the essential roles in ROS homeostasis and cell survival under stress conditions, NADK has been considered as a drug target in anti-cancer medicine research. Recently, two different strategies were used to restrain the growth of cancer cells by inhibiting NADK activity. Dihydrofolate reductase is an essential enzyme involved in *de novo* purine and thymidine biosynthesis but needs NADPH for the activity. Benzamide riboside, a drug targeted to NADK, can reduce NADP and NADPH levels, and therefore treatment with this drug can lead to the process of DNA synthesis and growth of cancer cells being inhibited (Roussel et al., 2012). Thionicotinamide adenine dinucleotide phosphate is also used as an inhibitor of NADK to accelerate the degradation of dihydrofolate reductase and thus to inhibit the growth of cancer cells (Hsieh et al., 2013). The other strategy is to inhibit proliferation by inducing high level of ROS in cancer cells. NADPH is essential for the neutralization of the dangerously high levels of ROS generated by increased metabolic activity of cancer cells. Inhibition of NADK activity with shRNA or thionicotinamide increased the ROS production in cancer cells which led to synergistic cell death (Tedeschi et al., 2015, 2016). These results demonstrate the value of NADK in anti-cancer research, and at the same time give us an attractive cue to use similar approaches for stress tolerance of plants.

## Conclusions and future perspectives

In plant cells, NADK family proteins are distributed in different subcellular compartments, including the cytosol, chloroplasts, and peroxisomes however, no NADK have been identified in mitochondria. Given that the mitochondrial respiratory chain can generate large amount of ROS, mitochondria become a major source of oxidative stress. In yeast and human cells, mitochondrial NADK plays an important role in maintaining redox balance. Thus, it is reasonable to believe that there may be an unknown NADK in mitochondria in plant cells although no homologous gene of human MNADK was found in plants. In addition, as discussed above, although many studies have shown that NADK plays crucial roles both in plant growth and development and stress tolerance, its cellular and molecular functions, and transcription regulatory mechanisms, are still under investigation. Considering their differential expression profiles in tissue and development specificity and environment dependence, NADK family genes may function in diverse aspects of plant growth and development. Further studies should be addressed to clarify the functional regulation and stress response of plant NADKs in cellular and molecular levels with considering both the paralogous members from same species and the orthologous from different species. More importantly, NADK-related applications in bacteria and animals enlighten us that plant NADKs may also have a great potential for developing plants with improved resistance to various biotic or abiotic stresses.

## Author contributions

B-BL and XW contributed equally to this work. B-BL and XW wrote the paper. LT and T-TM analyzed the data. W-TL, W-QL and AS revised the manuscript. K-MC provided funding and revised the manuscript.

### Conflict of interest statement

The authors declare that the research was conducted in the absence of any commercial or financial relationships that could be construed as a potential conflict of interest.

## Supplementary material

Figure S1 Developmental expression patterns of NADK family genes in plants. Expression profiles of nine plant NADK families at different developmental stages were obtained from the microarray data and mRNA sequence data reported by Genevestigator V3 (https://genevestigator.com/gv/). These NADK families include in four eudicotyledons plants, *AtNADK*s **(A)**, *SlNADK*s **(B)**, *GmNADK*s **(C)**, and *MtNADK*s **(D)**, and in four liliopsida plants, *OsNADK*s **(E)**, *HvNADK*s **(F)**, *ZmNADK*s **(G)**, *SbNADK*s **(H)**, and *TaNADK*s **(I)**. Results were shown as heat maps with white/gray/red (low to high) that reflect the percentages of the gene expression.

Figure S2 Anatomical expression patterns of NADK family genes in plants. Expression profiles of nine plant NADK family genes in different tissue anatomical structures were obtained from the microarray data and mRNA sequence data reported by Genevestigator V3 (https://genevestigator.com/gv/). These NADK gene families include in four eudicotyledons plants, *SlNADK*s **(A)**, *GmNADK*s **(B)**, *MtNADK*s **(E)**, and *AtNADK*s **(I)**, and in five liliopsida plants, *SbNADK*s **(C)**, *HvNADK*s **(D)**, *OsNADK*s **(F)**, *TaNADK*s **(G)**, and *ZmNADK*s **(H)**. Results were shown as heat maps with white/gray/red (low to high) that reflect the percent of expression.

Figure S3 Expression patterns of NADK family genes in eight plant species under abiotic and biotic stresses and hormone treatments. Expression profiles of eight plant NADK family genes were obtained from the microarray data and mRNA sequencing database data reported in Genevestigator V3 (https://genevestigator.com/gv/). These NADK gene families include in *Arabidopsis thaliana* **(A)**, *Oryza sativa* **(B)**, *Triticum aestivum* **(C,E)**, *Zea mays* **(D)**, *Hordeum vulgare* **(F)**, *Medicago truncatula* **(G)**, *Glycine max* **(H)**, and *Solanum lycopersicum* **(I)**. These profiles illustrate differential expression of NADK families from different plants under different abiotic and biotic stresses and hormone treatments including pathogen, cold, drought, heat, salt, submergence, ABA, GA3, IAA, JA-like, NAA, and SA. Results were shown as heat maps with green/dark/red which represent the indicated relative signal values; where greener represents stronger down-regulated expression and redder represents stronger up-regulated expression as compared to the controls.
