# Supplementary figures and images for: NAD Kinases: Metabolic Targets Controlling Redox Co-enzymes and Reducing Power Partitioning in Plant Stress and Development
Source: Front Plant Sci. 2018 Mar 23;9:379. doi: 10.3389/fpls.2018.00379 (PMC5890153; doi:10.3389/fpls.2018.00379)

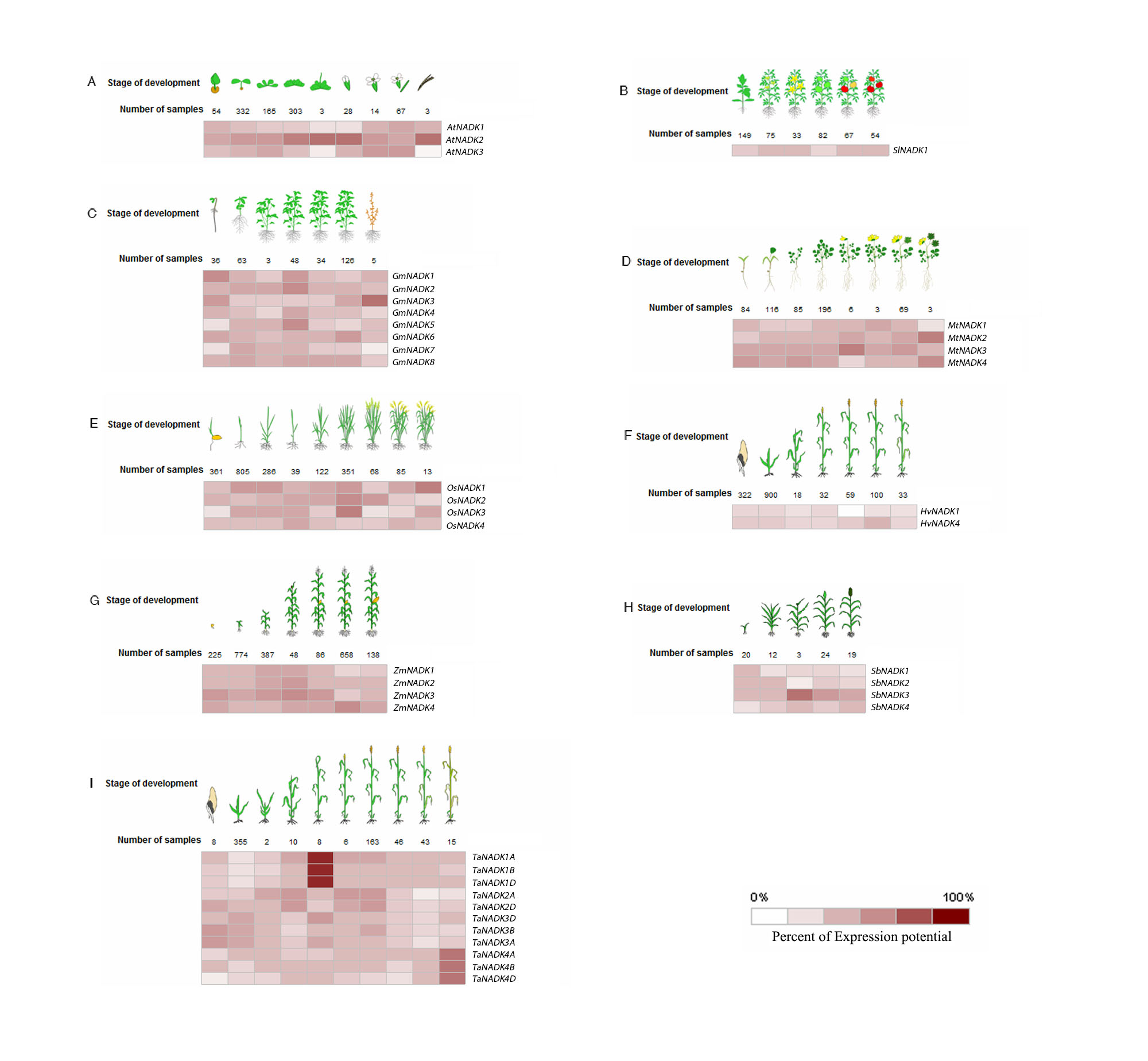

Supplement: Figure S1 — Developmental expression patterns of NADK family genes in plants. Expression profiles of nine plant NADK families at different developmental stages were obtained from the microarray data and mRNA sequence data reported by Genevestigator V3 (https://genevestigator.com/gv/). These NADK families include in four eudicotyledons plants, AtNADKs (A), SlNADKs (B), GmNADKs (C), and MtNADKs (D), and in four liliopsida plants, OsNADKs (E), HvNADKs (F), ZmNADKs (G), SbNADKs (H), and TaNADKs (I). Results were shown as heat maps with white/gray/red (low to high) that reflect the percentages of the gene expression. [file DataSheet1.zip › Supplementary Figures/Figure S1.JPEG]

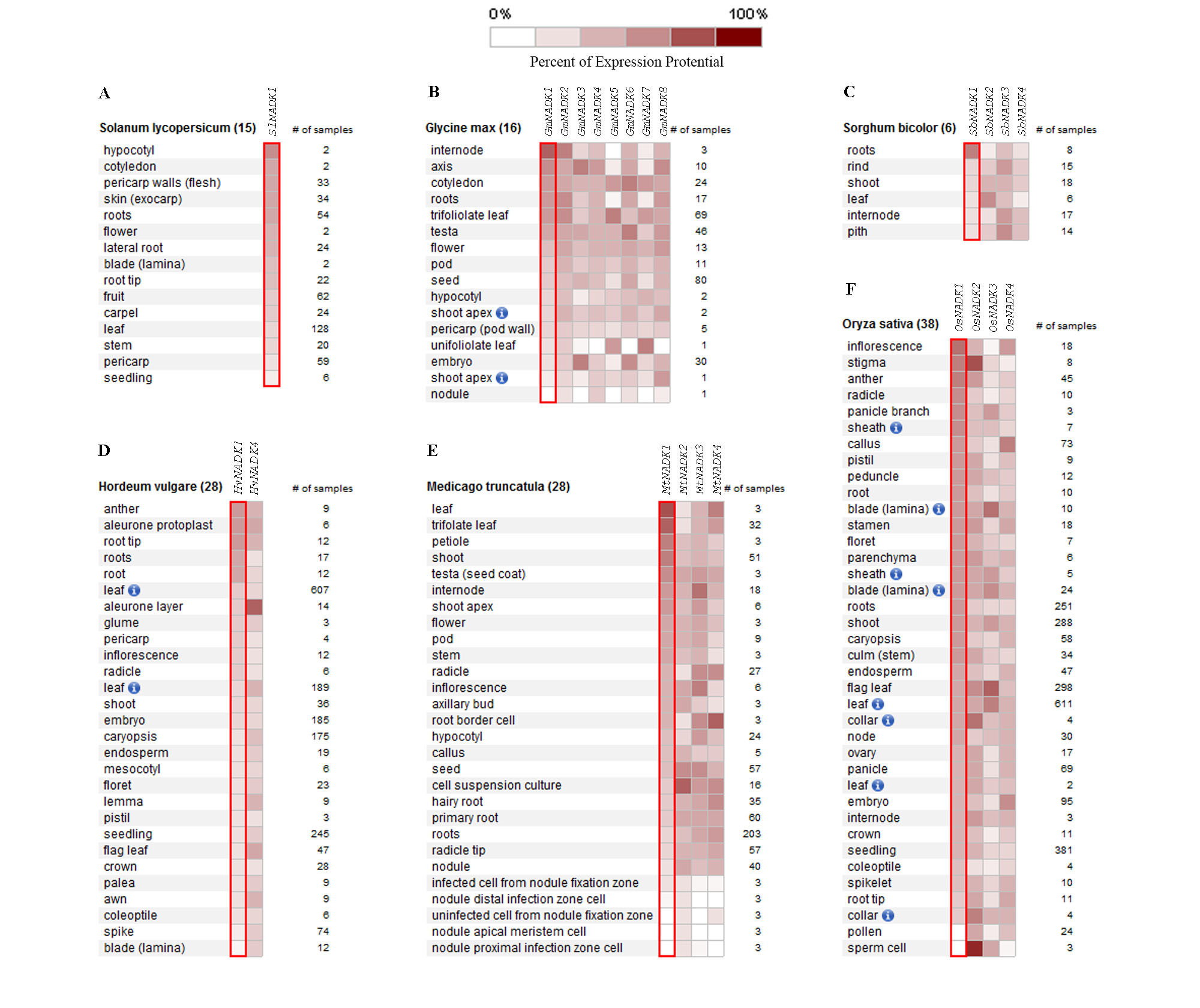

Supplement: Figure S1 — Developmental expression patterns of NADK family genes in plants. Expression profiles of nine plant NADK families at different developmental stages were obtained from the microarray data and mRNA sequence data reported by Genevestigator V3 (https://genevestigator.com/gv/). These NADK families include in four eudicotyledons plants, AtNADKs (A), SlNADKs (B), GmNADKs (C), and MtNADKs (D), and in four liliopsida plants, OsNADKs (E), HvNADKs (F), ZmNADKs (G), SbNADKs (H), and TaNADKs (I). Results were shown as heat maps with white/gray/red (low to high) that reflect the percentages of the gene expression. [file DataSheet1.zip › Supplementary Figures/Figure S2 (A-F).JPEG]

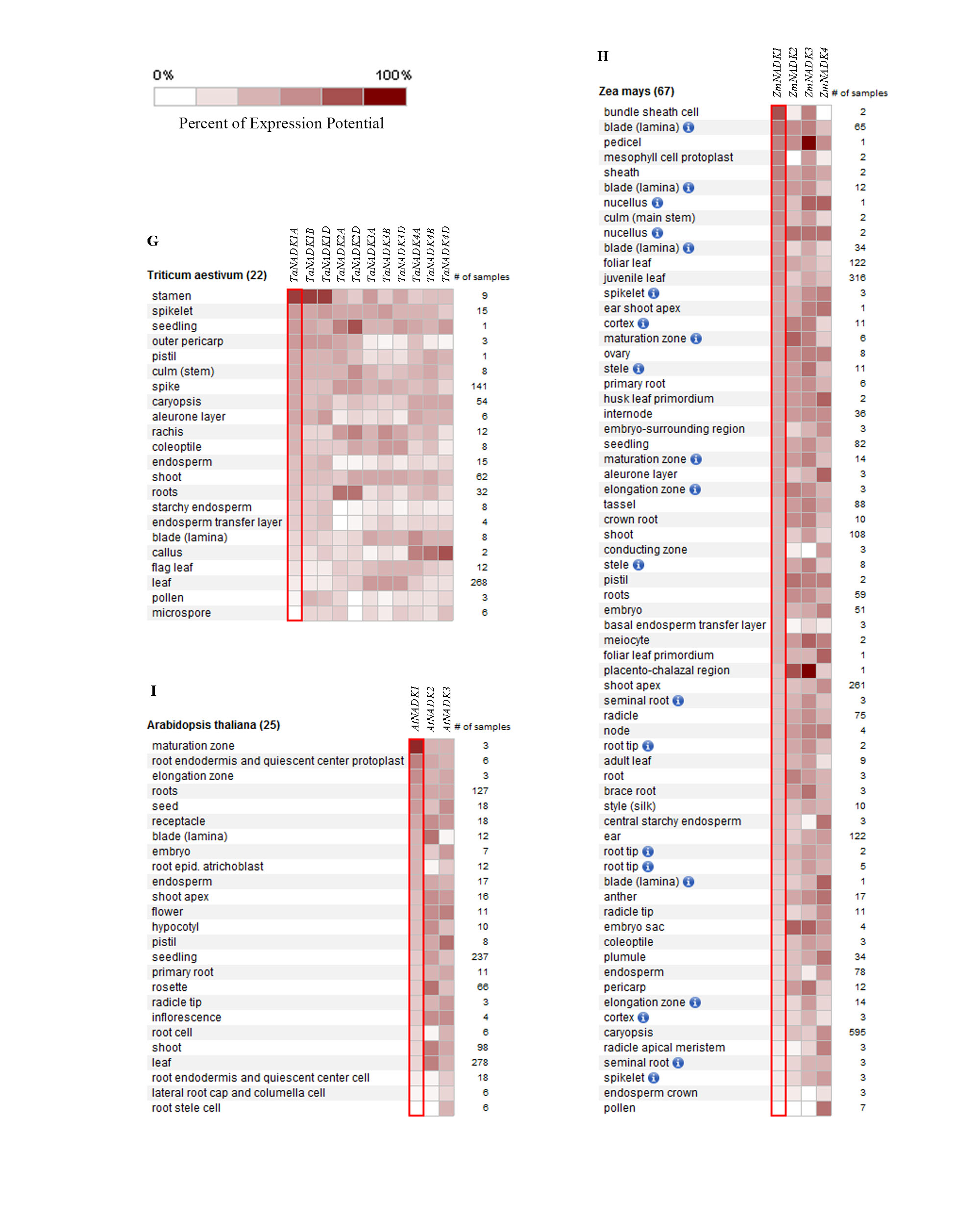

Supplement: Figure S1 — Developmental expression patterns of NADK family genes in plants. Expression profiles of nine plant NADK families at different developmental stages were obtained from the microarray data and mRNA sequence data reported by Genevestigator V3 (https://genevestigator.com/gv/). These NADK families include in four eudicotyledons plants, AtNADKs (A), SlNADKs (B), GmNADKs (C), and MtNADKs (D), and in four liliopsida plants, OsNADKs (E), HvNADKs (F), ZmNADKs (G), SbNADKs (H), and TaNADKs (I). Results were shown as heat maps with white/gray/red (low to high) that reflect the percentages of the gene expression. [file DataSheet1.zip › Supplementary Figures/Figure S2 (G-I).JPEG]

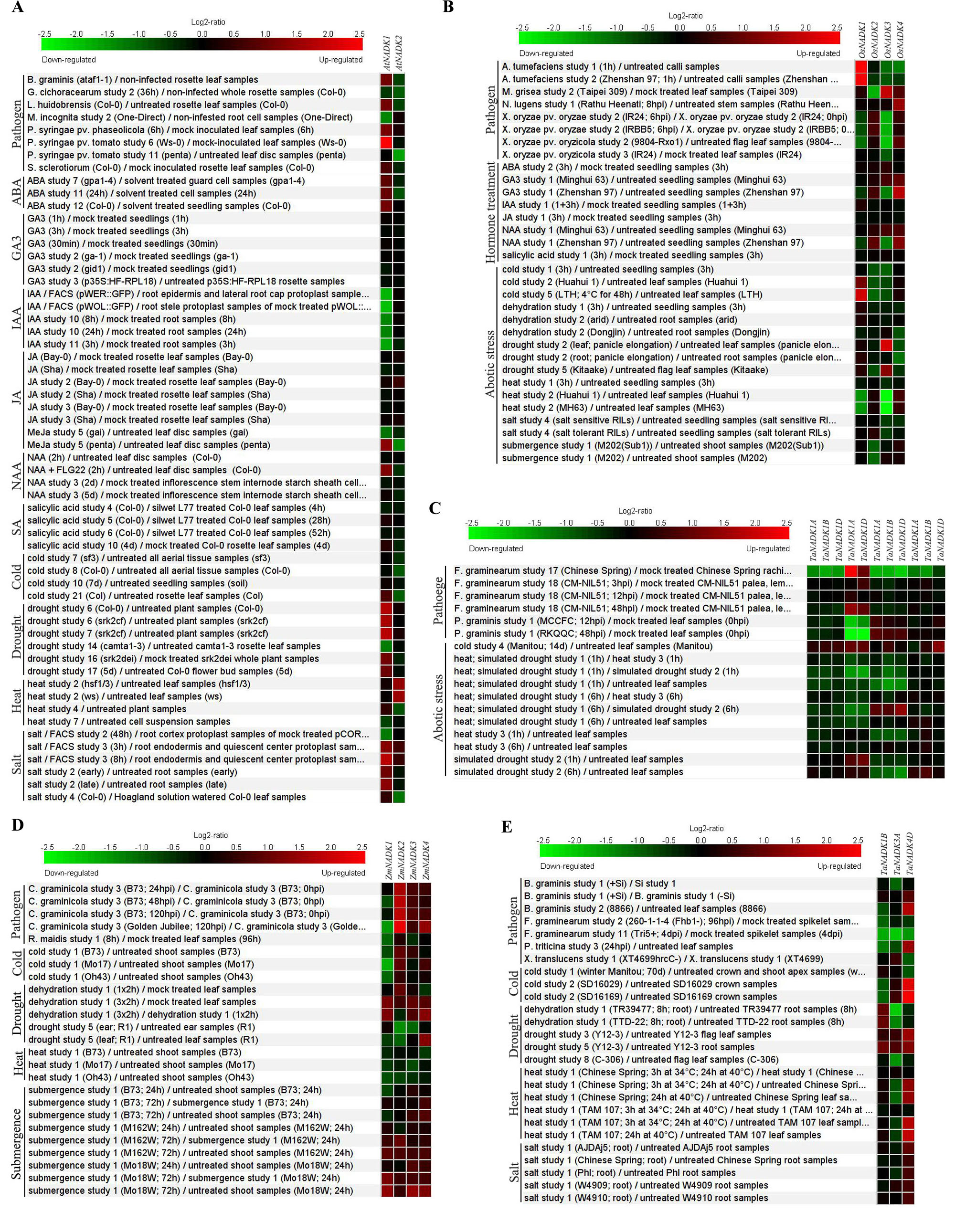

Supplement: Figure S1 — Developmental expression patterns of NADK family genes in plants. Expression profiles of nine plant NADK families at different developmental stages were obtained from the microarray data and mRNA sequence data reported by Genevestigator V3 (https://genevestigator.com/gv/). These NADK families include in four eudicotyledons plants, AtNADKs (A), SlNADKs (B), GmNADKs (C), and MtNADKs (D), and in four liliopsida plants, OsNADKs (E), HvNADKs (F), ZmNADKs (G), SbNADKs (H), and TaNADKs (I). Results were shown as heat maps with white/gray/red (low to high) that reflect the percentages of the gene expression. [file DataSheet1.zip › Supplementary Figures/Figure S3 (A-E).JPEG]

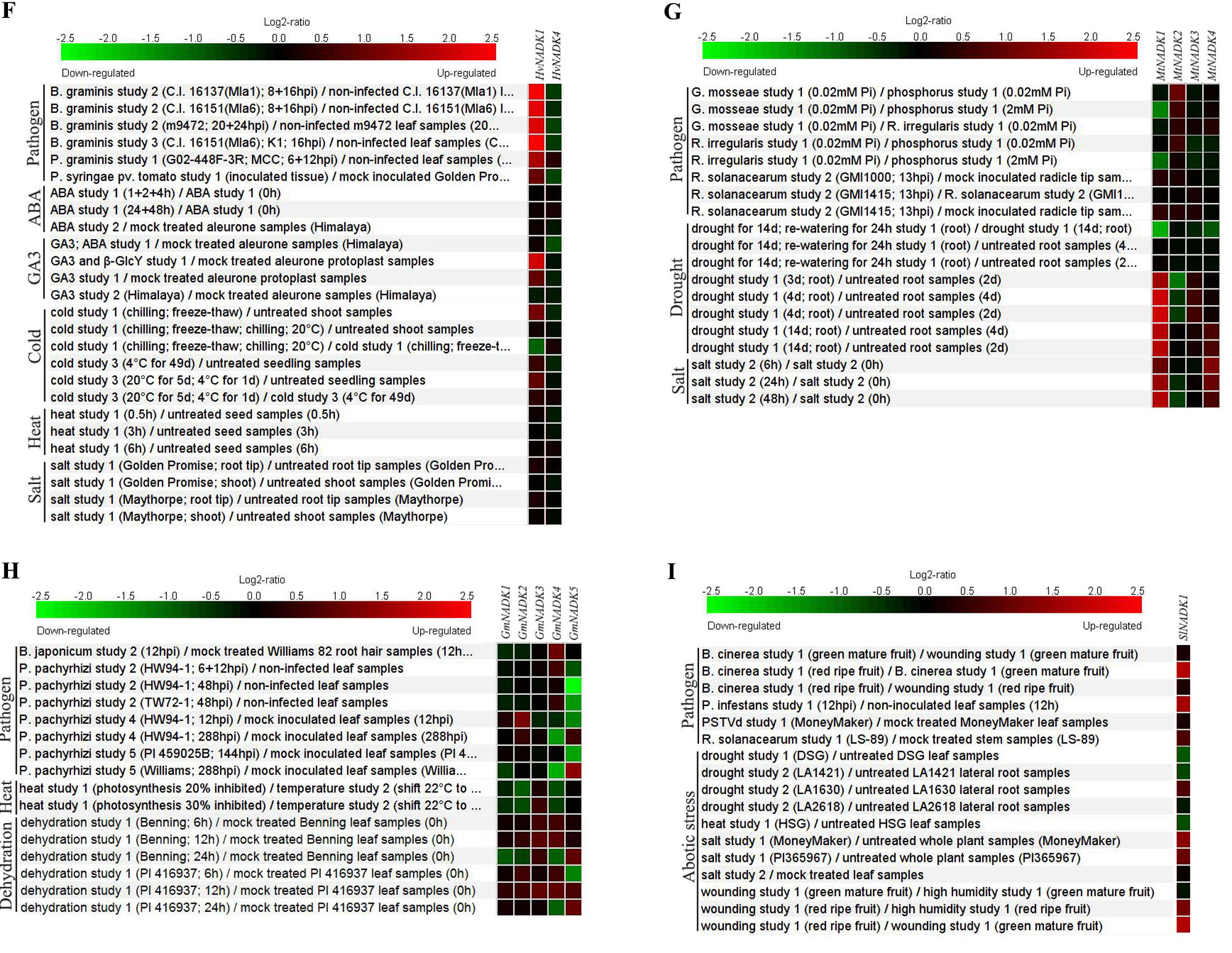

Supplement: Figure S1 — Developmental expression patterns of NADK family genes in plants. Expression profiles of nine plant NADK families at different developmental stages were obtained from the microarray data and mRNA sequence data reported by Genevestigator V3 (https://genevestigator.com/gv/). These NADK families include in four eudicotyledons plants, AtNADKs (A), SlNADKs (B), GmNADKs (C), and MtNADKs (D), and in four liliopsida plants, OsNADKs (E), HvNADKs (F), ZmNADKs (G), SbNADKs (H), and TaNADKs (I). Results were shown as heat maps with white/gray/red (low to high) that reflect the percentages of the gene expression. [file DataSheet1.zip › Supplementary Figures/Figure S3 (F-I).JPEG]
